# Exploration of circulating proteins associated with tumor stromal content using matched serum and tissue samples from patients with colon cancer

**DOI:** 10.1016/j.jlb.2026.100688

**Published:** 2026-10-23

**Authors:** F.(Floor) Heilijgers, J.E.(Fien) van Went, A.S.L.P.(Stijn) Crobach, C.M.(Christa) Cobbaert, K.C.M.J.(Koen) Peeters, L.R.(Renee) Ruhaak, B.J.M.(Bart) van Vlijmen, Y.E.M.(Yuri) van der Burgt, W.E.(Wilma) Mesker

**Affiliations:** a Department of Surgery, Leiden University Medical Center, Leiden, the Netherlands; b Department of Pathology, Leiden University Medical Center, Leiden, the Netherlands; c Department of Medicine, Division of Thrombosis and Hemostasis, Leiden University Medical Center, Leiden, the Netherlands; d Einthoven Laboratory for Vascular and Regenerative Medicine, Leiden University Medical Center, Leiden, the Netherlands; e Department of Clinical Chemistry and Laboratory Medicine, Leiden University Medical Center, Leiden, the Netherlands

**Keywords:** Colon cancer, Tumor microenvironment, Tumor-stroma ratio, Mass spectrometry, Serum proteins, Liquid biopsy

## Abstract

**Introduction:**

Liquid biopsies provide a source of biomolecules that allow tumor characterization. Increasing evidence shows that the tumor microenvironment (TME) plays a central role in behavior of colon cancer. This study assessed which proteins in the circulation correlate with tumor stromal content through analysis of serum and corresponding tissue samples from patients with colon cancer. A broad set of circulating proteins is quantified, ranging from common apolipoproteins, transport and complement factors, to key factors from interconnected stromal pathways such as coagulation and acute phase inflammation.

**Materials and methods:**

The cohort consisted of 97 patients with colon cancer and 50 control individuals who all underwent colonoscopy. Targeted mass spectrometry was applied to quantify 156 serum proteins via their surrogate peptides and corresponding internal standards. Matching solid tumor tissue slides from all 97 patients were used to determine the Tumor-Stroma Ratio (TSR). Serum protein levels were compared between stroma-high and stroma-low tumors, resulting in a subset of discriminating proteins. Composite protein scores were evaluated in multivariable Cox models.

**Results:**

Four serum proteins differed significantly between stroma-high and stroma-low tumors, all showing elevated levels in stroma-high patients, including complement-related proteins (C2, factor B, and C1r subcomponent-like protein) and the coagulation-related vitamin K-dependent protein S. This resulted in a composite Stromal Protein Score that may capture TME-driven variations. The Stromal Protein Score was significantly different between stroma-high and stroma-low patients (adjusted p < 0.001).

**Conclusion:**

Our data indicate that stroma-high tumors are associated with systemic alterations in complement and coagulation-related proteins. The results encourage further development of liquid biopsy–based protein profiling approaches, aiming for clinical implementation as a non-invasive strategy for TME-informed risk stratification and treatment monitoring in colon cancer.

## Highlights

• Stroma-high and stroma-low tumors show differences in serum proteins.
• Serum protein levels were associated with tumor stromal content.
• A Stromal Protein Score distinguished stroma-high from stroma-low tumors.
• The Stromal Protein Score showed no independent prognostic value for DFS.
• Liquid biopsy may enable minimally invasive assessment of tumor stroma.

## Abbreviations

ACT: Adjuvant Chemotherapy
AJCC: American Joint Committee on Cancer
CAF: Cancer-Associated Fibroblasts
CMS: Consensus Molecular Subtype
CV: Coefficients of Variation
DFS: Disease-Free Survival
ECM: Extracellular Matrix
FDR: False Discovery Rate
H&E: Hematoxylin and eosin
LC-MRM-MS: Liquid Chromatography Multiple Reaction Monitoring Mass Spectrometry
LUMC: Leiden University Medical Center
MSI: Microsatellite Instability
QC: Quality Control
SIL: Stable Isotope Labeled
TME: Tumor Microenvironment
TNM: Tumor Node Metastasis
TSR: Tumor-Stroma Ratio
UNITED: Uniform Noting for International application of Tumor-stroma ratio as Easy Diagnostic tool
UICC: Union for International Cancer Control

## 1 Introduction

Colon cancer is the third most prevalent cancer worldwide and a leading cause of cancer-related mortality [1]. The TNM (tumor-node-metastasis) classification remains an important framework for current treatment guidelines for colon cancer [2]. Additional pathological parameters that have been implemented in guidelines, such as Tumor Budding and Microsatellite Instability (MSI), mainly focus on the epithelial compartment of the tumor [3], [4]. Recent efforts within research regarding colon cancer have shifted towards the tumor microenvironment (TME). The TME comprises a complex dynamic ecosystem of blood vessels, the extracellular matrix (ECM) and non-neoplastic cells like fibroblasts and immune cells that surround and interact with tumor cells [5], [6]. These interactions shape fundamental aspects of tumor biology, influencing proliferation, invasion, metastatic potential, and evasion of the immune system. Moreover, accumulating evidence demonstrates that the composition and functional state of the TME strongly modulate treatment response, thereby affecting patient prognosis [5], [7].

The tumor stroma is a major component within the TME, and its abundance has been demonstrated to lead to worse patient-related outcomes [8], [9]. The Tumor Stroma Ratio (TSR) is a histopathological parameter based on the amount of stroma compared to the tumor epithelial component, expressed in percentages. Patients with >50% stroma are considered stroma-high patients and patients with ≤50% stroma are considered stroma-low patients [10]. The TSR has been shown to be of prognostic value for almost all epithelial cancers [11], [12]. The recent results of the UNITED study validated TSR as an independent prognosticator, confirming worse outcomes in patients with stroma-high colon cancer. Furthermore, patients with stroma-high colon cancer potentially experienced chemotherapy resistance as they demonstrated no benefit from adjuvant chemotherapy (ACT) [13]. This significant result paved the way inclusion of TSR in the latest edition of the TNM-classification by The Union for International Cancer Control (UICC) and the American Joint Committee on Cancer (AJCC) [14].

In parallel, molecular classifications have provided additional insight into the biological significance of the tumor stroma. The Consensus Molecular Subtype (CMS) classification system identifies four subtypes based on the tumor stroma, characterized by a high content of stromal fibroblasts (CAFs) [15]. CMS4 tumors show the highest risk for developing distant metastasis, a feature that appears to be driven predominantly by stromal biology rather than intrinsic epithelial characteristics [16], [17].

Building on these advances in stromal and molecular tumor characterization, additional approaches have emerged to further refine tumor assessment.

One such method is tumor characterization through molecular analysis of liquid biopsies [18]. Liquid biopsy refers to the examination of biological fluids from cancer patients to detect tumor-derived biomarkers [19]. A principal advantage of this approach is its minimally invasive nature, which allows sampling to be performed repeatedly throughout disease progression and treatment. Consequently, liquid biopsies have been proposed as a valuable method for early detection and longitudinal biomarker monitoring [20], [21]. In colon cancer, this technique may provide a more comprehensive representation of overall tumor heterogeneity compared with conventional solid tissue biopsy [22]. Despite these promising features, the TME has remained underappreciated in current liquid biopsy research. Ravensbergen et al. (2021) demonstrated that a stromal–epithelial gene signature ratio derived from single-cell transcriptomics combined with proteomics data predicts survival in colon adenocarcinoma. Furthermore, protein signatures may reflect histologic intratumoral stromal content and hence provide valuable prognostic information in liquid biopsy [18]. A recent study by Buijs et al. (2026) identified serum proteins associated with venous thromboembolism in patients with colorectal cancer [23].

In the present study, we therefore applied the serum protein panel identified by Buijs et al. [24] to investigate whether these circulating proteins may also reflect differences in tumor stromal content in colon cancer. The aim of this study was to evaluate whether serum protein quantification can discriminate patients with stroma-high and stroma-low colon cancer and thereby capture characteristics of the TME. The overarching objective is to develop a liquid biopsy–based assay as an alternative or complementary approach to tissue biopsy in colon cancer for characterization and prognostication with future perspectives for monitoring treatment response.

## 2 Materials and methods

### 2.1 Patient cohort

Serum samples were obtained from the biobank of the Leiden University Medical Center (LUMC) and originated from participants in the Dutch National Colorectal Cancer Screening Program. The study cohort comprised 97 patients with colon cancer and 50 individuals without colon cancer, enrolled between 2015 and 2020. Controls, defined as patients without colon cancer, were individuals who had been referred for colonoscopy following a positive fecal occult blood test result but were subsequently found to have no abnormalities during colonoscopy. Patients with stage I–IV disease who underwent curative-intent surgical resection of the primary tumor were included, with some receiving ACT. Included patients with stage IV disease had liver metastases that were surgically treated after resection of the primary tumor in a second-stage procedure. All patients were treated at the LUMC. The study protocol was reviewed and approved by the scientific committee of the LUMC. All blood samples were collected at the patient's first hospital visit, prior to surgery or colonoscopy. For patients who provided multiple blood samples at different time points the first available sample was used for subsequent analyses to ensure that the samples reflected the patients' baseline disease status.

### 2.2 Quantification of 156 serum proteins using multiple reaction monitoring mass spectrometry

Serum proteins were quantified using liquid chromatography multiple reaction monitoring mass spectrometry (LC-MRM-MS) assay. To this end, serum proteins were first converted into peptides in a standard bottom-up proteomics workup procedure, followed by measurement of one quantifier peptide (surrogate) for each protein that was targeted. Moreover, for each quantifier peptide a synthetic stable isotope-labeled (SIL) peptide was added as internal standard allowing accurate determination of a relative peptide quantity. This ratio of endogenous to SIL peptide correlates with the quantity of the target protein. The ratios were standardized to a pooled citrate plasma calibrator that was included in the total measurement procedure.

In total 156 serum proteins were targeted and quantified, with a selection that was based on previous studies [25], [26], [27]. The list is shown in Supplementary Table 1. These proteins span diverse functional domains, including 21 coagulation-related proteins, 18 complement proteins, 15 transport-related proteins and 14 apolipoproteins according to annotation in the Human Protein Atlas [28], [29].

Semi-automated sample preparation, including protein digestion, was performed using a BRAVO Liquid Handling System (Agilent Technologies, Santa Clara, USA). Peptides were purified through solid-phase extraction, separated using a reversed-phase LC column (EclipsePlusC18 length and internal diameter 150 × 2.1 mm, 1.8 μm particles; Agilent Technologies) and analyzed on a triple-quadrupole mass spectrometer (Agilent 6495C; Agilent Technologies) operating in MRM mode.

Simultaneously with measurement of the cohort the performance of the LC-MRM-MS assay was evaluated from ten independent runs of citrate plasma pools and two quality control (QC) serum samples. Although metrics on assay performance are specific for the type of matrix used, for the majority of proteins a similar performance was observed in serum or plasma. An obvious difference between these matrices is the absence of fibrinogen alpha/beta/gamma chains in sera. Assay performance in the two QC samples demonstrated coefficients of variation (CVs) below 15% for 146 proteins of which 123 proteins were even below 7% (Supplementary Table 2).

### 2.3 TSR assessment

Hematoxylin and eosin (H&E)-stained tissue slides of the primary tumor were obtained from patients with colon cancer, preferably from the most invasive region of the tumor, as indicated in the pathologist's report [10]. For the ten patients with T1 tumors, no residual tumor tissue was found in the resection specimen, as it had already been removed via biopsy. In these cases, biopsy specimens were used to determine TSR, which has previously been shown to be prognostic in rectal cancer [30]. TSR was scored by two clinical researchers (FH and FEW) following the protocol described by Van Pelt et al. (2018) [10]. In cases of uncertainty or when the TSR percentage was near 50%, the slide was reviewed by a third researcher (WEM). If uncertainty persisted, the slide could be referred to a pathologist (ASLPC) for further review. All researchers had completed the official TSR e-learning, and FH and WEM demonstrated high inter-observer agreement in a previous study [24], [31]. Supplementary Fig. 1 shows an example of a stroma-low and a stroma-high tumor.

### 2.4 Statistical analysis

Statistical analysis was carried out using IBM SPSS Statistics for Windows, version 29.0 (IBM Corp., Armonk, NY) and R statistical software, version 4.5.1. (R Foundation for Statistical Computing, Vienna, Austria). Protein distributions were assessed using the Shapiro-Wilk test. Differences in individual serum protein levels between patients with stroma-high and stroma-low colon cancer were evaluated using Mann-Whitney U tests for non-normally distributed proteins. To control for multiple comparisons, p-values were adjusted using the Benjamini-Hochberg False Discovery Rate (FDR), with Q-values < 0.05 considered significant [32]. To capture the combined effect of all significant proteins, a z-score was calculated for each protein per individual and averaged across all significant proteins to generate a ‘Stromal Protein Score’. This protein score was then compared between patients with stroma-high and stroma-low colon cancer [33]. Individual serum proteins were further analyzed between patients with colon cancer and controls, applying the same statistical approach.

A survival analysis was conducted in the colon cancer subgroup. Multivariable Cox proportional hazards models were used to assess the prognostic value of the Stromal Protein Score while adjusting for established clinical prognostic factors. The primary outcome was disease-free survival (DFS), defined as the time from surgery to recurrence, defined as distant metastasis or locoregional recurrence, or death from any cause. Patients were censored if they were disease-free and/or alive at the last follow-up or registration date. Results are reported as hazard ratios with 95% confidence intervals and corresponding p-values. Median follow-up time was calculated using reversed Kaplan-Meier analysis.

## 3 Results

### 3.1 Clinical characteristics of patients with colon cancer

The study cohort included 147 individuals, of whom 97 were diagnosed with colon cancer. Baseline characteristics of patients with colon cancer are summarized in Table 1. The median age of patients with colon cancer was 63 years, and 60 patients (61.9%) were male. Most tumors were located in the sigmoid colon (n = 41; 42.3%), with 52 patients (53.6%) undergoing a sigmoidectomy. Postoperatively, 16 patients (16.5%) experienced complications. The cohort comprised 27 (27.8%) stage I, 31 (32.0%) stage II, 36 (37.1%) stage III, and 3 (3.1%) stage IV patients. TSR could not be assessed in 12 patients (12.4%) due to insufficient tissue in biopsy samples or inadequate quality of the available pathology slides. Stroma-high tumors were observed in 30 patients (30.9%). Of all patients with colon cancer, 31 (32.0%) received adjuvant chemotherapy (ACT). During follow-up, 16 patients (16.5%) developed distant metastases, including two with concurrent locoregional recurrence. At the end of follow-up, 12 patients (12.4%) had died. The median follow-up time was five years.

**Table 1:** Baseline characteristics of patients in the patients with colon cancer in cohort.

Baseline characteristics	Colon cancer patient cohort (N = 97)
Gender	
Male	60 (61.9)
Female	37 (38.1)

Age at surgery	63 (59 – 68)
Median age	
Location of tumor	
Coecum	7 (7.2)
Colon ascendencs	11 (11.3)
Flexura hepatica	5 (5.2)
Colon transversum	12 (12.4)
Flexura lienalis	5 (5.2)
Colon descendens	3 (3.1)
Sigmoid	41 (42.3)
Unknown	13 (13.4)

Surgery type	
Hemicolectomy right	29 (29.9)
Hemicolectomy left	13 (13.4)
Sigmoidectomy	52 (53.6)
Other^a^	3 (3.1)

Complication^b^	
Yes	16 (16.5)
No	79 (81.4)
Unknown	2 (2.3)

Pathological T-stage^c^	
T1	15 (15.5)
T2	20 (20.6)
T3	54 (55.7)
T4	8 (8.2)

Pathological N-stage^c^	
N0	58 (59.8)
N1	29 (29.9)
N2	10 (10.3)

Pathological TNM stage^c^	
Stage I	27 (27.8)
Stage II	31 (32.0)
Stage III	36 (37.1)
Stage IV	3 (3.1)

Tumor morphology	
Adenocarcinoma	91 (93.8)
Mucinous carcinoma	6 (6.2)

Tumor-Stroma Ratio	
Stroma-high (>50%)	30 (30.9)
Stroma-low (≤50%)	55 (56.7)

Adjuvant chemotherapy received	
Yes	31 (32.0)
No	66 (68.0)

Adjuvant chemotherapy regimen^d^	
CAPOX	27 (87.1)
Capecitabine	4 (12.9)

Recurrence	
Total number of events	16 (16.5)
Distant metastasis	16 (16.5)
Locoregional recurrence and distant metastasis	2 (2.1)

Mortality	12 (12.4)
Total number of deaths	

All variables are given as absolute numbers with associated percentages or medians with interquartile ranges. Sum of percentages can be less or more than 100 due to rounding.

TNM, tumor–node–metastasis.

a Other operation types include a (sub)total colectomy, high anterior resection, or transversectomy.

b Complications included hemorrhage, anastomotic leakage and wound infection.

c The versions of the Union for International Cancer Control (UICC) TNM classification at time of inclusion were used.

d Shown as percentage of total number of patients that received adjuvant chemotherapy.

### 3.2 Univariable analysis of serum protein panel between stroma-high and stroma low patients

The sample grouping in the unsupervised hierarchical clustering showed no batch effect regarding origin (Supplementary Fig. 2). To adjust for multiple testing, the Benjamini–Hochberg FDR procedure was applied. A volcano plot was subsequently generated to visualize these results (Fig. 1A). After correction, four proteins showed significant differences between patients with stroma-high and stroma-low colon cancer. These proteins are shown in Fig. 2. These four significantly altered proteins were further evaluated in controls. Kruskal–Wallis testing demonstrated significant overall differences in circulating protein levels among controls, patients with stroma-low, and stroma-high colon cancer for all four proteins, indicating that at least one group differed significantly for each protein. These findings are shown in Fig. 3. Post hoc Dunn's tests with Bonferroni correction showed that complement factor B levels were higher in stroma-high patients than in both controls (adjusted p = 0.0017) and stroma-low patients (adjusted p = 0.0007), with no difference between controls and stroma-low patients (adjusted p = 1.00). Complement C2 levels differed between controls and stroma-low patients (adjusted p = 0.008) and between stroma-low and stroma-high patients (adjusted p < 0.001), but not between controls and stroma-high patients (adjusted p = 0.546). Vitamin K–dependent protein S levels differed between stroma-high and stroma-low patients (adjusted p = 0.005), with no significant differences between the other groups. Overall, these findings suggest that circulating levels of several proteins are associated with stromal phenotype, with differences observed between stroma-high and stroma-low tumors for several proteins. Given the previously established prognostic relevance of a stroma-high phenotype, these findings warrant further investigation of circulating proteins as potential markers of tumor–stroma characteristics.

**Figure 1:**
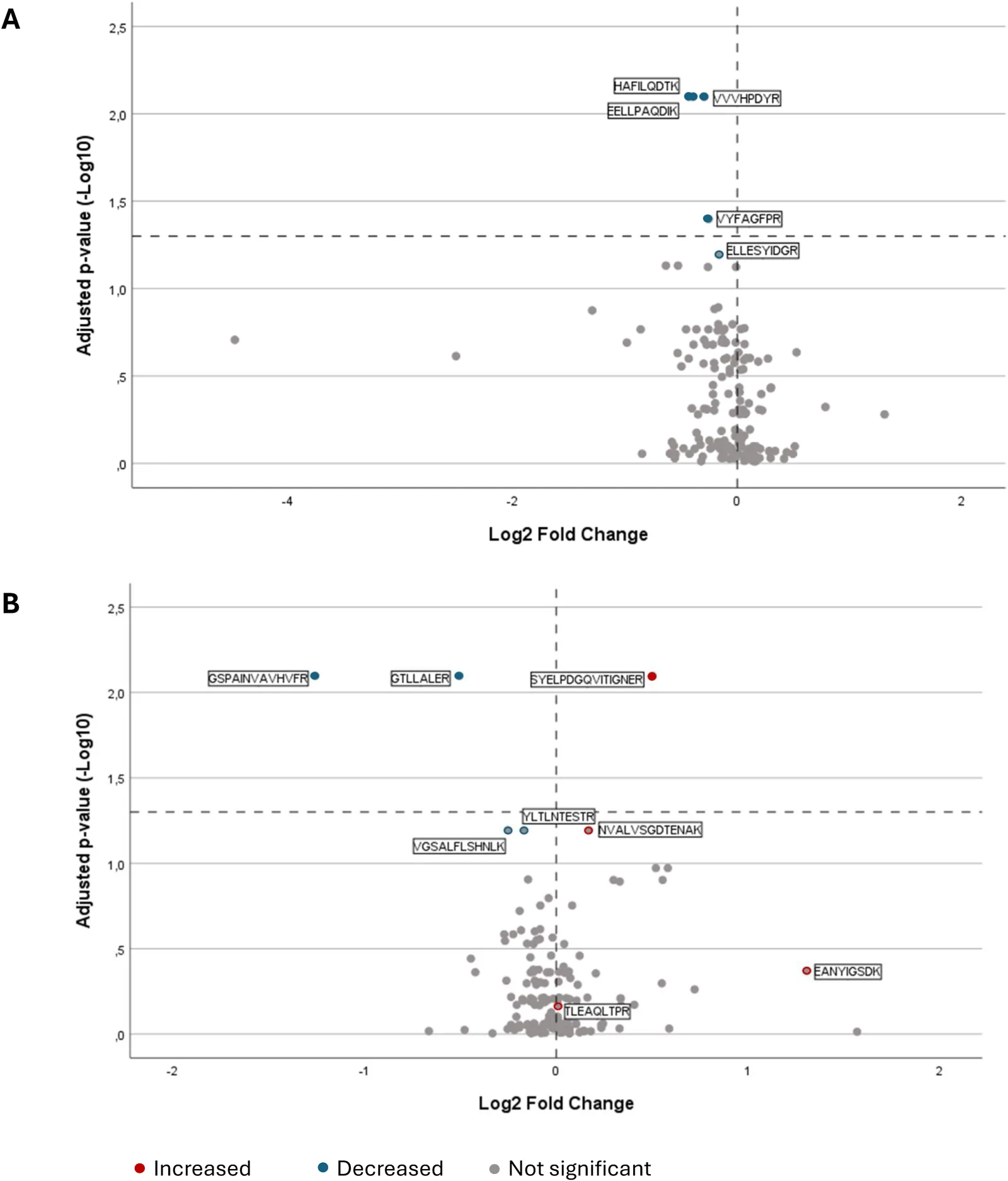
Top discriminating proteins in cohort. A) A volcano-plot of all measured proteins within patients with colon cancer with x-axis representing log2 foldchange with stroma-low patients as reference, and y-axis representing –log10 P-value of non-parametric Wilcoxon rank-sum test. B) A volcano-plot of all measured proteins in full cohort with x-axis representing log2 foldchange with controls as reference, and y-axis representing –log10 P-value of non-parametric Wilcoxon rank-sum test.

**Fig. 2:**
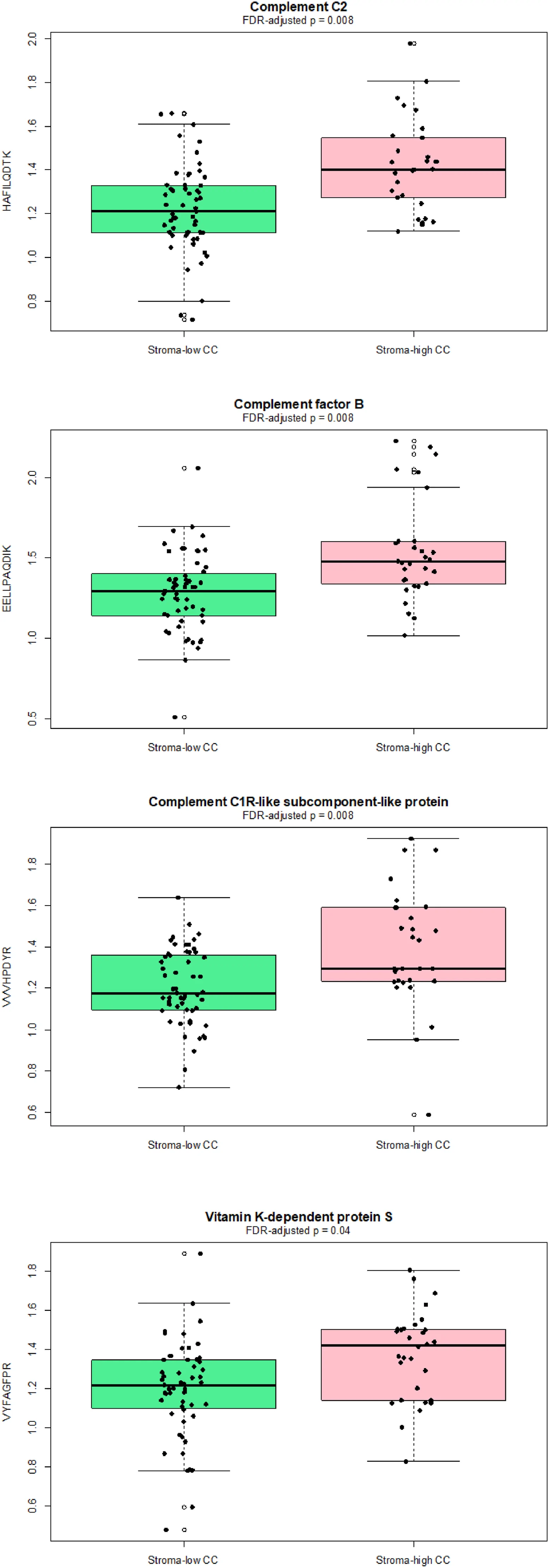
Univariable analysis of top discriminative protein levels within the patients with colon cancer, comparing stroma-high and stroma-low tumors. Boxplots with serum protein levels on y-axis demonstrating a significant difference in proteins with diverse secretory functions. Notably, all significant proteins were elevated in stroma-high patients and originated from circulating proteins involved in complement activation and coagulation pathways. Complement-related proteins included complement C2 (HAFILQDTIK), complement factor B (EELPAQDIK), and complement C1r subcomponent-like protein (VVVHPDYR), while coagulation-associated proteins comprised vitamin K–dependent protein S (VYFAGFPR). CC = colon cancer; FDR = false discovery rate.

**Fig. 3:**
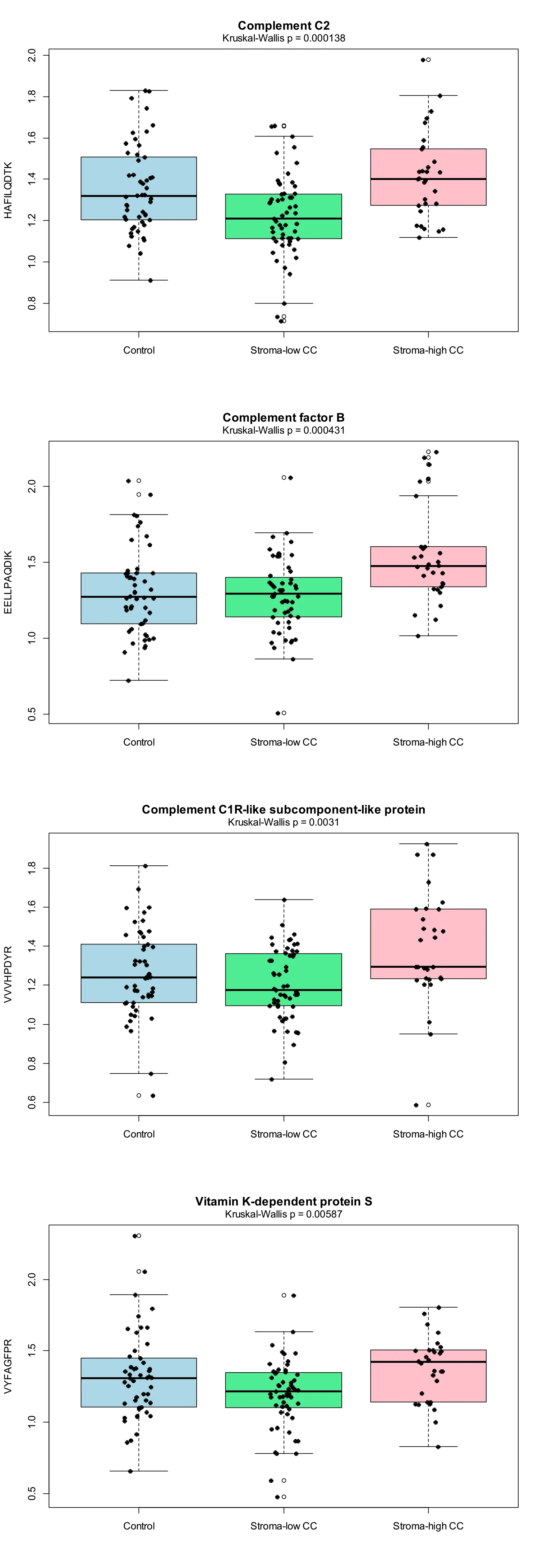
Univariable analysis of top discriminative protein levels, comparing stroma-high and stroma-low tumors with controls. Boxplots with serum protein levels on y-axis demonstrating a significant difference in proteins with diverse secretory functions. Notably, all significant proteins were elevated in stroma-high patients and originated from circulating proteins involved in complement activation and coagulation pathways. Kruskal–Wallis testing demonstrated significant overall differences in protein levels among controls, patients with stroma-low, and stroma-high colon cancer for all four proteins, indicating that at least one group differed significantly for each marker. Comparable levels between controls and stroma-low patients were seen. These findings suggest that circulating levels of several proteins are associated with stromal phenotype, with differences observed between stroma-high and stroma-low tumors. CC = colon cancer.

### 3.3 Calculation of Stromal Protein Score

Thereafter, the Stromal Protein Score was calculated as the mean z-score of the above-mentioned proteins. This score was compared within the colon cancer subgroup between stroma-high and stroma-low patients. Stroma-high demonstrated a higher Stromal Protein Score compared to stroma-low patients (p < 0.001). The discriminatory performance of the Stromal Protein Score for distinguishing patients with stroma-high from stroma-low patients was subsequently assessed using receiver operating characteristic (ROC) curve analysis. The area under the ROC curve (AUC) was 0.70 (95% CI 0.54–0.81). Using the Youden index, the optimal cutoff was −0.14, corresponding to a sensitivity of 80% and specificity of 55%. This is shown in Supplementary Fig. 3. Subsequently, the Stromal Protein Score was compared among controls, patients with stroma-low, and stroma-high colon cancer. Stromal Protein Score levels in stroma-low patients were comparable to those observed in controls, whereas stroma-high patients showed more elevated scores. These findings are shown in Fig. 4. Post hoc Dunn's tests with Bonferroni correction showed that the Stromal Protein Score was significantly different between stroma-high and stroma-low patients (adjusted p < 0.001), whereas no significant difference was observed between controls and stroma-high or stroma-low patients.

**Fig. 4:**
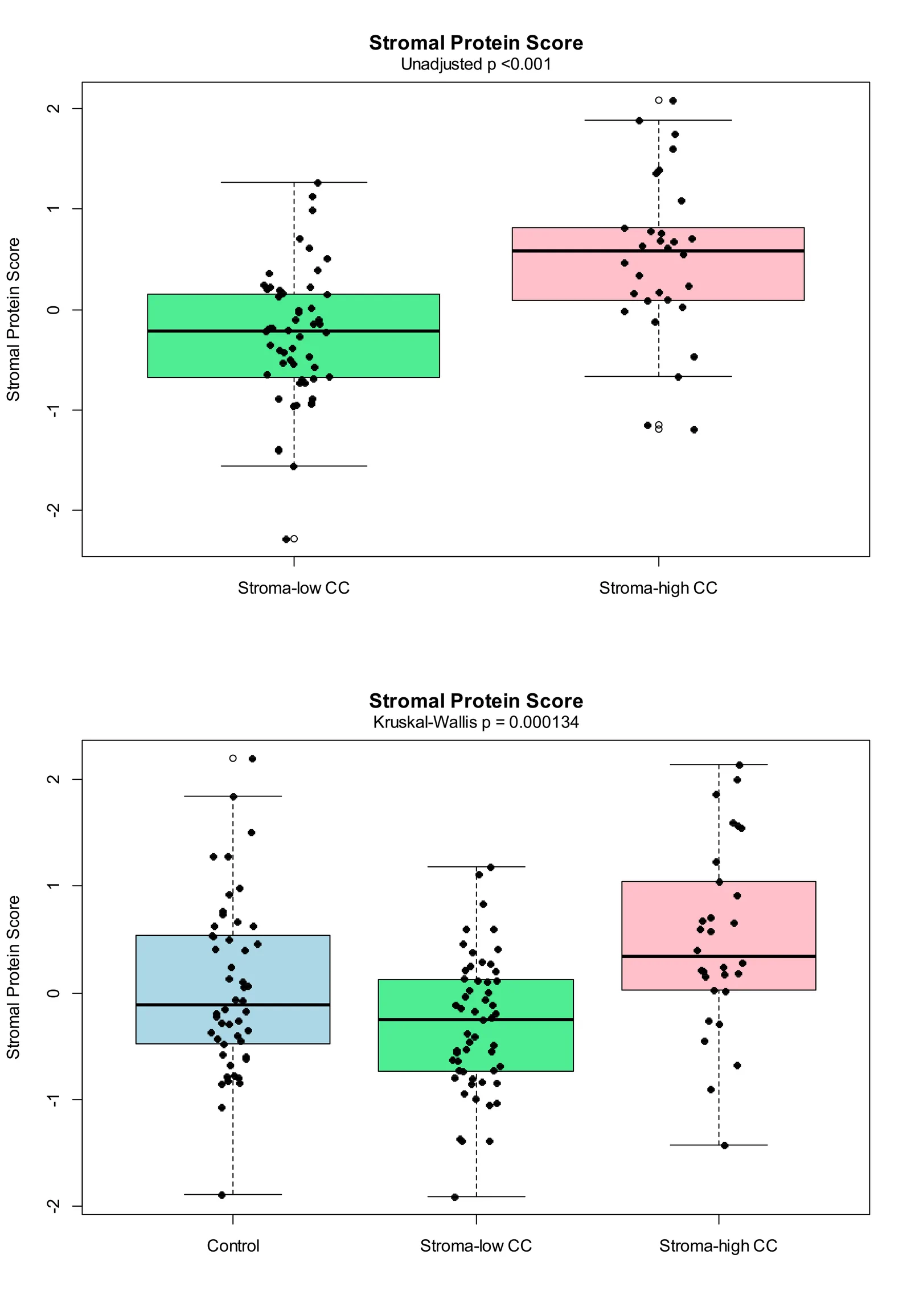
A boxplot showing analysis in the patients with colon cancer, demonstrating a significant difference in the Stromal Protein Score between stroma-high and stroma-low tumors. The Stromal Protein Score was calculated as the mean z-score of the above-mentioned proteins. This score was compared within the colon cancer subgroup between stroma-high and stroma-low patients. Stroma-high demonstrated a higher Stromal Protein Score compared to stroma-low patients. Subsequently, the Stromal Protein Score was compared among controls, patients with stroma-low, and stroma-high colon cancer. Stromal Protein Score levels in stroma-low patients were comparable to those observed in controls, whereas stroma-high patients showed elevated scores. CC = colon cancer.

Since the Stromal Protein Score showed significant differences within the colon cancer subgroup, it was further evaluated in multivariable Cox regression analysis. To address potential collinearity between the Stromal Protein Score and TSR, two separate multivariable models were performed: one including both the Stromal Protein Score and TSR, and a second model excluding TSR. The Stromal Protein Score did not demonstrate independent prognostic value for DFS in either model. The results of the models are presented in Supplementary Table 3A and 3B

### 3.4 Univariable analysis of protein panel between patients with colon cancer and controls

As an additional analysis, differences in protein levels between controls and patients with colon cancer were visualized. A volcano plot was generated (Fig. 1B), and after FDR correction, six proteins showed significant differential expression between patients with colon cancer and controls. These proteins are presented in Supplementary Fig. 4. Thereafter, the Diagnostic Protein Score was calculated as the mean z-score of the above-mentioned proteins. This score was compared between patients with colon cancer and controls and showed a significant difference, with higher values in controls. However, the magnitude of this difference was small and no further post-hoc Dunn's test was performed. These findings are shown in Supplementary Fig. 5.

## 4 Discussion

In this exploratory study, we assessed differences in circulating protein levels between patients with stroma-high and stroma-low colon cancer using a combination of LC-MRM-MS-based serum protein quantification and pathological analysis of corresponding tumor tissues. Notably, all significant proteins were elevated in stroma-high patients and originated from circulating proteins involved in complement activation and coagulation pathways. Proteins that differed significantly represented proteins with diverse secretory functions, with larger effect sizes than those observed when comparing protein levels between controls and patients with colon cancer. Complement-related proteins included complement C2 (HAFILQDTIK), complement factor B (EELPAQDIK), and complement C1r subcomponent-like protein (VVVHPDYR), while coagulation-associated proteins comprised vitamin K–dependent protein S (VYFAGFPR) [29]. Together, these results indicate an association with higher tumor stroma content and increased serum levels of proteins linked to immune and hemostatic processes. However, this exploratory study demonstrates an association but cannot fully establish causality between TSR and serum protein levels as potential confounding factors, such as systemic inflammation or comorbidities. Could not be fully accounted for.

The Stromal Protein Score, calculated as the mean z-score of the above-mentioned proteins, suggested stromal subgroups by showing higher values in stroma-high disease. Furthermore, Stromal Protein Score levels in stroma-low patients were comparable to those observed in controls. This may indicate that the composite score captures subtle systemic protein variations, reflecting biologically relevant differences related to TME composition rather than general case–control variation. This hypothesis is further supported by the functional characteristics of contributing proteins. All proteins driving this score originate from circulating proteins involved in complement activation and coagulation, pathways that are closely linked to stromal biology, ECM remodeling, inflammation, and tumor–host interactions [34], [35].

The complement system, a key part of immunity, consists of serum proteins that opsonize pathogens and trigger inflammatory responses, aiding immune defense and maintaining homeostasis [36], [37]. Within the TME, stromal, endothelial and immune cells contribute to local production of complement proteins. Evidence indicates that complement activity within the TME can have dual roles, either promoting or inhibiting tumor progression, although human data remains limited and occasionally contradictory [38]. A stroma-high TME is known to be associated with decreased immune cell infiltration and fibroblast activation, processes that can systemically influence complement signaling. Locally produced complement components interact with ECM and stromal signals, shaping immune responses and tumor biology. In particular, fibroblasts with activated signaling pathways can secrete complement factors that modulate macrophage polarization, contributing to the immunosuppressive remodeling of the TME. Consistent with this, our study observed elevated complement levels in stroma-high tumors, highlighting the significant contribution of the dense stromal compartment.

The hemostatic system, beyond its role in coagulation, interacts dynamically with the TME. Stromal fibroblasts, endothelial cells, immune cells, and platelets contribute to local production and activation of coagulation factors [35], [39]. Hemostatic activity in the TME can enhance angiogenesis, modulate immune cell recruitment, and influence tumor progression in a context-dependent manner. A stroma-high TME is associated with increased interactions among activated fibroblasts, platelets, and coagulation factors, processes that can systemically affect hemostatic signaling [35], [40]. Locally generated hemostatic components interact with the ECM and stromal cues, shaping immune responses and vascular remodeling [41], [42]. In line with this, our study observed elevated hemostatic pathway activity in stroma-high tumors, highlighting the contribution of the dense stromal compartment.

However, the targeted nature of the 156-protein panel should be considered when interpreting these findings. This protein panel was originally selected based on its association with venous thromboembolism risk in colorectal cancer rather than to provide unbiased coverage of the circulating proteome. Consequently, the relatively high representation of complement- and coagulation-related proteins in the panel may have predisposed the analysis toward identifying differences in these pathways. Thus, although the identified proteins are biologically plausible in relation to stromal biology, the predominance of complement- and coagulation-related proteins may reflect the predefined composition of the panel rather than specific associations with tumor stromal content. Future studies that include additional protein targets could help establish whether these associations extend beyond the pathways represented in the current panel and provide a more complete picture of the circulating protein signature associated with tumor stromal content.

The processes in a stroma-rich TME can systemically influence complement and hemostatic signaling [35], [43]. The predominance of proteins within the Stromal Protein Score therefore suggests that it captures downstream systemic manifestations of stromal activity rather than tumor-cell–intrinsic signals. This biological coherence strengthens the hypothesis that the score reflects TME-driven physiology. However, it also explains why its prognostic value may be limited, as these pathways are not specific to tumor aggressiveness but instead represent broader host response mechanisms. This is further reflected in the survival analyses, in which no significant prognostic association of the Stromal Protein Score with DFS was observed. Considering the relatively small and clinically heterogeneous patient cohort, the multivariate model may be overfitted and the absence of a strong prognostic signal was not unexpected.

In a secondary analysis, differences in serum protein levels between patients with colon cancer and controls were evaluated. Although six analyzed peptides reached statistical significance, the magnitude of these differences was small. Collectively, no robust serum protein alterations distinguishing patients with colon cancer from controls were identified. However, this comparison, which is primarily aimed at cancer detection, was considered beyond the scope of the present analysis, particularly given the limited sample size.

A key strength of this study is the standardized sampling and handling of serum specimens. All samples were derived from the biobank at the LUMC, ensuring uniform collection, processing, and storage procedures and thereby minimizing pre-analytical variability. In addition, the control group consisted of individuals with a positive fecal occult blood test who were subsequently found not to have colorectal cancer on colonoscopy, which improved comparability with cases and reduces sampling heterogeneity. Furthermore, all patients were diagnosed and managed within the same clinical setting, reducing variability in diagnostic work-up and clinical procedures.

However, the single-center design may limit external validity and the relatively small sample size may restrict the generalizability of the findings. In addition, heterogeneity in the patient population should be considered, as colon cancer stages ranged from I to IV. Prior literature suggests that tumor biology differs substantially across stages, with a greater dependence on stromal components in earlier-stage disease compared to metastatic stages [44], [45], [46], [47], [48]. Consequently, it is difficult to determine whether the observed differences are attributable to stromal content itself or to differences in disease stage or tumor burden. To assess the potential influence of stage IV disease, we repeated the univariate analyses after excluding the three patients with stage IV colon cancer. This did not change the direction of the associations or their statistical significance for any of the individual proteins or the Stromal Protein Score. Furthermore, circulating complement- and coagulation-related proteins may be influenced by systemic inflammation, comorbidities, anticoagulant treatment, and tumor burden, independently of tumor stromal content. Due to the limited sample size, we were unable to perform adequately powered multivariable analyses to adjust for these potential confounding factors. Consequently, we cannot exclude the possibility that the observed associations between serum protein levels and TSR were partly attributable to these factors. Larger studies are needed to assess the independent association between circulating protein levels and tumor stromal content while accounting for relevant clinicopathological and patient-related variables. However, due to the limited sample size in this study, subgroup analyses were underpowered and did not allow for these specific conclusions. Lastly, the lack of separate discovery and validation cohorts is a limitation. Future studies are therefore warranted to enable subgroup analyses to further elucidate stage-dependent differences and validation of the Stromal Protein Score in an independent cohort, preferably in a multicenter setting.

Future perspectives should further explore whether the identified protein panel can provide information on TME-related features in colon cancer. Expanding and refining these protein signatures may help to better characterize the biological heterogeneity of the TME minimally invasive sampling. Although the discriminatory performance of the Stromal Protein Score was moderate, the data presented is clinically relevant by providing complementary, minimally invasive protein information on the TME. However, the present study did not demonstrate an association between the Stromal Protein Score and DFS, and its prognostic or predictive value therefore remains to be established. Further validation in larger, independent cohorts will be required to determine whether these biomarkers provide clinically relevant information beyond established clinicopathological factors and whether they may ultimately have a role in personalized patient management.

## 5 Conclusion

Our data showed that stroma-high and stroma-low tumors show differences in serum proteins and that stromal content within tumors showed more pronounced systemic alterations in complement and coagulation-related proteins. Composite protein scores may capture these subtle biological variations, with the Stromal Protein Score reflecting TME-driven physiology. The results encourage further development of liquid biopsy–based protein profiling approaches, aiming for clinical implementation as a non-invasive strategy for TME-informed risk stratification and treatment monitoring in colon cancer in the future.

## Ethics declaration

Written informed consent to take part in the study and to publish the article has been obtained from all participants or their legal representatives. The privacy rights of participants have been observed.

This study included organ or tissue donors. This study includes human biological material and consent was obtained by donors, or their next of kin or legal representatives, for use in this study and for publication of the article. The samples used in this research were not sourced from executed prisoners or prisoners of conscience.

This study was performed in compliance with relevant laws, regulatory frameworks and guidelines where the research took place. This study was approved by the METC LUMC. (Approval No. -)

## Data availability statement

The data that support the findings of this study are available on request from the corresponding author. The data are not publicly available due to privacy or ethical restrictions.

## Ethics statement

All patients were treated at the LUMC. The study protocol was reviewed and approved by the scientific committee of the LUMC. All patients gave informed consent as part of the LUMC Biobank.

## Author contributions

F.H. executed data processing and data analysis. F.H. and J.E.W wrote the study protocol and submitted this to the biobank of the LUMC. J.E.W. and Y.E.M.B. sampled the serum of the included patients. F.H. and J.E.W. sampled the H&E slides and corresponding clinical data. Scoring TSR on the H&E slides was done by F.H., J.E.W., W.E.M. and A.S.L.P.C. Laboratory analyses were performed by B.J.V.M. and Y.E.M.B and, together with L.R.R., provided expertise in proteomics. F.H., K.C.M.J.P, A.S.L.P.C. and W.E.M. provided expertise in colon cancer histopathology and biology. F.H. wrote the first draft of the manuscript which was later revised and approved by all co-authors. W.E.M. provided overall supervision of the project.

## Disclosure

All authors report no financial or non-financial conflict of interest.

## Declaration of competing interest

The authors declare that they have no known competing financial interests or personal relationships that could have appeared to influence the work reported in this paper.

## Figures and Tables

**figs1:**
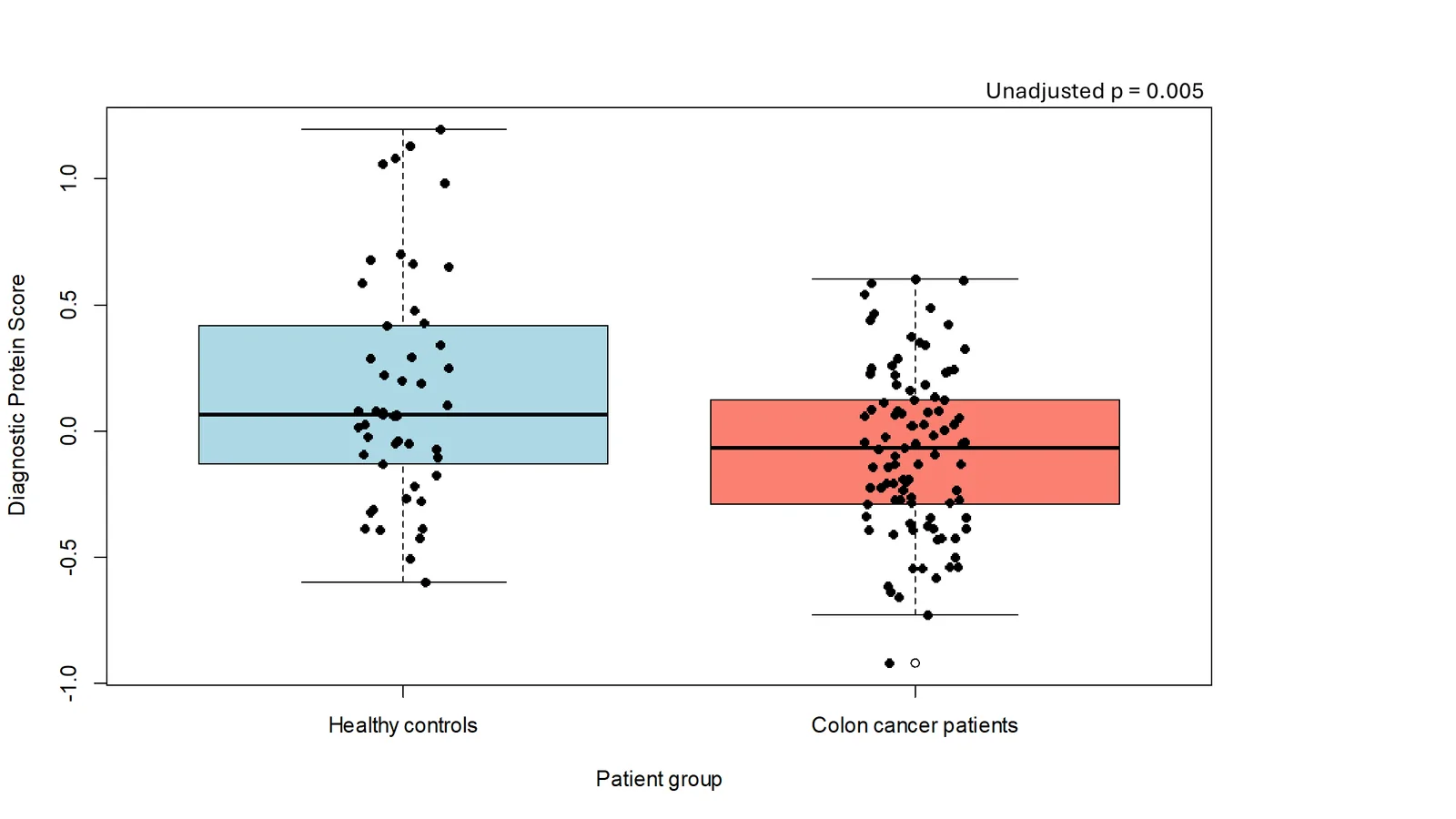


## Appendix A Supplementary data

Fig. S1 Showing examples of colon tumors in study cohort. A) Demonstrating a stroma-high tumor (90% intratumoral stroma) and B) demonstrating a stroma-low tumor (10% intratumoral stroma).

Fig. S2 Overview of quantified protein concentrations in patient serum in the study cohort. The heatmap with clustering shows all quantified proteins in columns and samples in rows along with clustering of both. The sample clinical data are represented in the annotation map on the left.

Fig. S3 Receiver operating characteristic (ROC) curve analysis demonstrating he discriminatory performance of the Stromal Protein Score for distinguishing patients with stroma-high from stroma-low colon cancer. The area under the ROC curve (AUC) was 0.70 (95% CI 0.54–0.81). Using the Youden index, the optimal cutoff was −0.14, corresponding to a sensitivity of 80% and specificity of 55%.

Fig. S4 Univariable analysis of top discriminative protein levels in the full cohort, comparing patients with colon cancer with controls. Boxplots with serum protein level on y-axis demonstrating a significant difference in six proteins, although the magnitude of these differences was small. The significant peptides represented proteins with diverse secretory functions. Actin, aortic smooth muscle (SYELPDGQVITIGNER) and extracellular matrix protein 1 (NVALVSGDTENAK) showed slightly higher levels in patients with colon cancer, whereas thrombospondin-1 (GTLLALER), transthyretin (GSPAINVAVHVFR), and cholinesterase (YLTLNTESTR) were modestly higher in controls. Serum amyloid A1/A2 (VGSALFLSHNLK) was statistically different but displayed a negligible absolute difference. Collectively, these findings indicate that while minor statistical differences were detected, no robust serum protein alterations distinguishing patients with colon cancer from controls were identified.

## References

[1] Koncina E., Haan S., Rauh S., Letellier E. (2020). Prognostic and predictive molecular biomarkers for colorectal cancer: updates and challenges. Cancers (Basel).

[2] Brierley J., Gospodarowicz M.K., Wittekind C. (2017).

[3] Lugli A., Kirsch R., Ajioka Y., Bosman F., Cathomas G., Dawson H. (2017). Recommendations for reporting tumor budding in colorectal cancer based on the International Tumor Budding Consensus Conference (ITBCC) 2016. Mod Pathol.

[4] Roth A.D., Delorenzi M., Tejpar S., Yan P., Klingbiel D., Fiocca R. (2012). Integrated analysis of molecular and clinical prognostic factors in stage II/III colon cancer. J Natl Cancer Inst.

[5] Anderson N.M., Simon M.C. (2020). The tumor microenvironment. Curr Biol.

[6] Zhang H., Yue X., Chen Z., Liu C., Wu W., Zhang N. (2023). Define cancer-associated fibroblasts (CAFs) in the tumor microenvironment: new opportunities in cancer immunotherapy and advances in clinical trials. Mol Cancer.

[7] Li H., Fan X., Houghton J. (2007). Tumor microenvironment: the role of the tumor stroma in cancer. J Cell Biochem.

[8] Mesker W.E., Junggeburt J.M., Szuhai K., de Heer P., Morreau H., Tanke H.J. (2007). The carcinoma-stromal ratio of colon carcinoma is an independent factor for survival compared to lymph node status and tumor stage. Cell Oncol.

[9] Mesker W.E., Liefers G.J., Junggeburt J.M., van Pelt G.W., Alberici P., Kuppen P.J. (2009). Presence of a high amount of stroma and downregulation of SMAD4 predict for worse survival for stage I-II colon cancer patients. Cell Oncol.

[10] van Pelt G.W., Kjær-Frifeldt S., van Krieken J., Al Dieri R., Morreau H., Tollenaar R. (2018). Scoring the tumor-stroma ratio in colon cancer: procedure and recommendations. Virchows Arch.

[11] Vangangelt K.M.H., Green A.R., Heemskerk I.M.F., Cohen D., van Pelt G.W., Sobral-Leite M. (2020). The prognostic value of the tumor-stroma ratio is most discriminative in patients with grade III or triple-negative breast cancer. Int J Cancer.

[12] Smit M.A., Philipsen M.W., Postmus P.E., Putter H., Tollenaar R.A., Cohen D. (2020). The prognostic value of the tumor-stroma ratio in squamous cell lung cancer, a cohort study. Cancer Treat Res Commun.

[13] Polack M., Smit M.A., van Pelt G.W., Roodvoets A.G.H., Meershoek-Klein Kranenbarg E., Putter H. (2024). Results from the UNITED study: a multicenter study validating the prognostic effect of the tumor–stroma ratio in colon cancer. ESMO Open.

[14] Brierley J.D., Giuliani M., O'Sullivan B., Rous B., Eycken E.V. (2025).

[15] Ten Hoorn S., de Back T.R., Sommeijer D.W., Vermeulen L. (2022). Clinical value of consensus molecular subtypes in colorectal cancer: a systematic review and meta-analysis. J Natl Cancer Inst.

[16] Guinney J., Dienstmann R., Wang X., de Reyniès A., Schlicker A., Soneson C. (2015). The consensus molecular subtypes of colorectal cancer. Nat Med.

[17] Sandberg T.P., Oosting J., van Pelt G.W., Mesker W.E., Tollenaar R.A.E.M., Morreau H. (2018). Molecular profiling of colorectal tumors stratified by the histological tumor-stroma ratio - increased expression of galectin-1 in tumors with high stromal content. Oncotarget.

[18] Ravensbergen C.J., Kuruc M., Polack M., Crobach S., Putter H., Gelderblom H. (2021). The Stroma liquid Biopsy Panel contains a stromal-epithelial gene signature Ratio that is associated with the histologic tumor-stroma Ratio and predicts survival in Colon cancer. Cancers (Basel).

[19] Yamada T., Matsuda A., Koizumi M., Shinji S., Takahashi G., Iwai T. (2019). Liquid biopsy for the management of patients with colorectal cancer. Digestion.

[20] Herrera M., Galindo-Pumariño C., García-Barberán V., Peña C. (2019). A snapshot of the tumor microenvironment in colorectal cancer: the liquid biopsy. Int J Mol Sci.

[21] Normanno N., Cervantes A., Ciardiello F., De Luca A., Pinto C. (2018). The liquid biopsy in the management of colorectal cancer patients: current applications and future scenarios. Cancer Treat Rev.

[22] Diederiks N., Ravensbergen C.J., Treep M., van Wezel M., Kuruc M., Renee Ruhaak L. (2023). Development of Tier 2 LC-MRM-MS protein quantification methods for liquid biopsies. J Mass Spectrom Adv Clin Lab.

[23] Buijs J.T., van Es N., Englisch C., Anijs R.J.S., Bosch F.T.M., van Vlijmen B.J.M. (2026). Absolute quantitative proteomics identifies patterns of plasma proteins associated with venous thromboembolism in patients with colorectal cancer. Thromb Res.

[24] Franken I., Heilijgers F., Bakker M.-C., Rubio-Alarcon C., Crnovrsanin N., Lemmens M. (2025). Combined prognostic value of post-surgery circulating tumor DNA and tumor-stroma ratio in patients with stage III colon cancer treated with adjuvant chemotherapy. J Clin Oncol.

[25] Guman N.A.M., Kraaijpoel N., Mulder F.I., Carrier M., Jara-Palomares L., Di Nisio M. (2025). Quantitative targeted proteomics for occult cancer screening in patients with unprovoked venous thromboembolism: results from the prospective PLATO-VTE study. Res Pract Thromb Haemost.

[26] Mohammed Y., Touw C.E., Nemeth B., van Adrichem R.A., Borchers C.H., Rosendaal F.R. (2022). Targeted proteomics for evaluating risk of venous thrombosis following traumatic lower-leg injury or knee arthroscopy. J Thromb Haemostasis.

[27] van der Vliet B.F., Camilleri E., Paes T., Treep M., Dolezal N., Cordfunke R.A. (2026). Distinct plasma protein profiles after long-term remission of Cushing's disease. J Clin Endocrinol Metabol.

[28] Uhlén M., Fagerberg L., Hallström B.M., Lindskog C., Oksvold P., Mardinoglu A. (2015). Proteomics. Tissue-based map of the human proteome. Science.

[29] Uhlén M., Karlsson M.J., Hober A., Svensson A.S., Scheffel J., Kotol D. (2019). The human secretome. Sci Signal.

[30] Polack M., van Pelt G.W., van den Heuvel D.H., Klein-Kranenbarg E.M., Roodvoets A.G.H., Putter H. (2025). The tumour-stroma ratio as predictive aid towards a biopsy-based treatment strategy in rectal carcinoma. Histopathology.

[31] Smit M.A., van Pelt G.W., Dequeker E.M., Al Dieri R., Tollenaar R.A., van Krieken J.H.J. (2021). e-Learning for instruction and to improve reproducibility of scoring tumor-stroma ratio in Colon carcinoma: performance and reproducibility assessment in the UNITED Study. JMIR Form Res.

[32] Benjamini Y., Hochberg Y. (2018). Controlling the false discovery rate: a practical and powerful approach to multiple testing. J Roy Stat Soc B.

[33] Morrison L., Laukkanen J.A., Ronkainen K., Kurl S., Kauhanen J., Toriola A.T. (2016). Inflammatory biomarker score and cancer: a population-based prospective cohort study. BMC Cancer.

[34] Balduit A., Agostinis C., Bulla R. (2025). Beyond the norm: the emerging interplay of complement system and extracellular matrix in the tumor microenvironment. Semin Immunol.

[35] Cantrell R., Palumbo J.S. (2022). Hemostasis and tumor immunity. Research and Practice in Thrombosis and Haemostasis.

[36] Merle N.S., Church S.E., Fremeaux-Bacchi V., Roumenina L.T. (2015). Complement system part I - molecular mechanisms of activation and regulation. Front Immunol.

[37] Merle N.S., Noe R., Halbwachs-Mecarelli L., Fremeaux-Bacchi V., Roumenina L.T. (2015). Complement system part II: role in immunity. Front Immunol.

[38] Roumenina L.T., Daugan M.V., Petitprez F., Sautès-Fridman C., Fridman W.H. (2019). Context-dependent roles of complement in cancer. Nat Rev Cancer.

[39] Chen L., Zhu C., Pan F., Chen Y., Xiong L., Li Y. (2023). Platelets in the tumor microenvironment and their biological effects on cancer hallmarks. Front Oncol.

[40] Obermann W.M.J., Brockhaus K., Eble J.A. (2021). Platelets, constant and cooperative companions of sessile and disseminating tumor cells, crucially contribute to the tumor microenvironment. Front Cell Dev Biol.

[41] Galmiche A., Rak J., Roumenina L.T., Saidak Z. (2022). Coagulome and the tumor microenvironment: an actionable interplay. Trends Cancer.

[42] Schaffner F., Ruf W. (2009). Tissue factor and PAR2 signaling in the tumor microenvironment. Arterioscler Thromb Vasc Biol.

[43] van Pelt G.W., Sandberg T.P., Morreau H., Gelderblom H., van Krieken J., Tollenaar R. (2018). The tumour-stroma ratio in Colon cancer: the biological role and its prognostic impact. Histopathology.

[44] Puccini A., Berger M.D., Zhang W., Lenz H.J. (2017). What we know about stage II and III Colon cancer: it's still not enough. Targeted Oncol.

[45] Roberto M., Arrivi G., Pilozzi E., Montori A., Balducci G., Mercantini P. (2022). The potential role of genomic signature in stage II relapsed colorectal cancer (CRC) patients: a mono-institutional study. Cancer Manag Res.

[46] Henry L.R., Lee H.O., Lee J.S., Klein-Szanto A., Watts P., Ross E.A. (2007). Clinical implications of fibroblast activation protein in patients with Colon cancer. Clin Cancer Res.

[47] Dariya B., Aliya S., Merchant N., Alam A., Nagaraju G.P. (2020). Colorectal cancer biology, diagnosis, and therapeutic approaches. Crit Rev Oncog.

[48] Furuhashi S., Bustos M.A., Mizuno S., Ryu S., Naeini Y., Bilchik A.J. (2023). Spatial profiling of cancer-associated fibroblasts of sporadic early onset Colon cancer microenvironment. npj Precis Oncol.

