# Supplementary material for: Exploration of circulating proteins associated with tumor stromal content using matched serum and tissue samples from patients with colon cancer
Source: J Liq Biopsy. 2026 Oct 23;14:100688. doi: 10.1016/j.jlb.2026.100688 (PMC13734802; doi:10.1016/j.jlb.2026.100688)
Supplement: Supplementary file 2 [file mmc2.pdf]

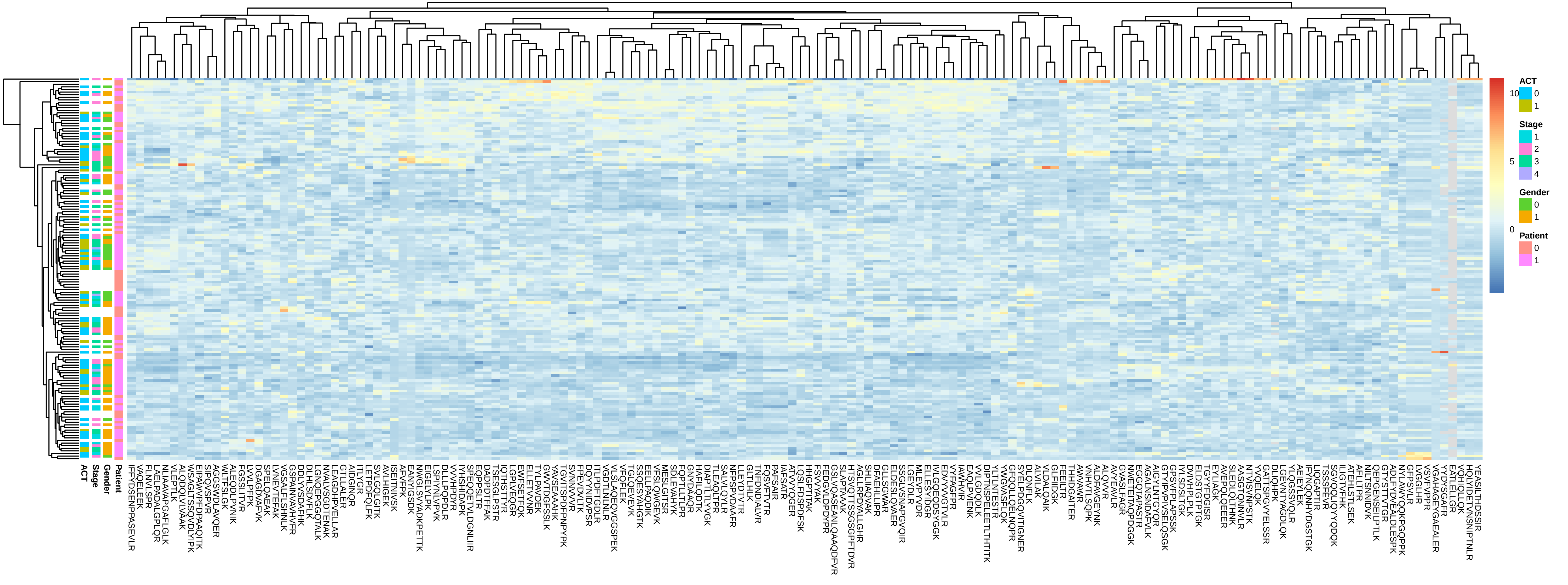
ACT
Stage
Gender
Patient
10
5
0
ACT
0
1
Stage
1
2
3
4
Gender
0
1
Patient
0
1
