# Supplementary material for: Exploration of circulating proteins associated with tumor stromal content using matched serum and tissue samples from patients with colon cancer
Source: J Liq Biopsy. 2026 Oct 23;14:100688. doi: 10.1016/j.jlb.2026.100688 (PMC13734802; doi:10.1016/j.jlb.2026.100688)
Supplement: Supplementary file 3 [file mmc3.pdf]

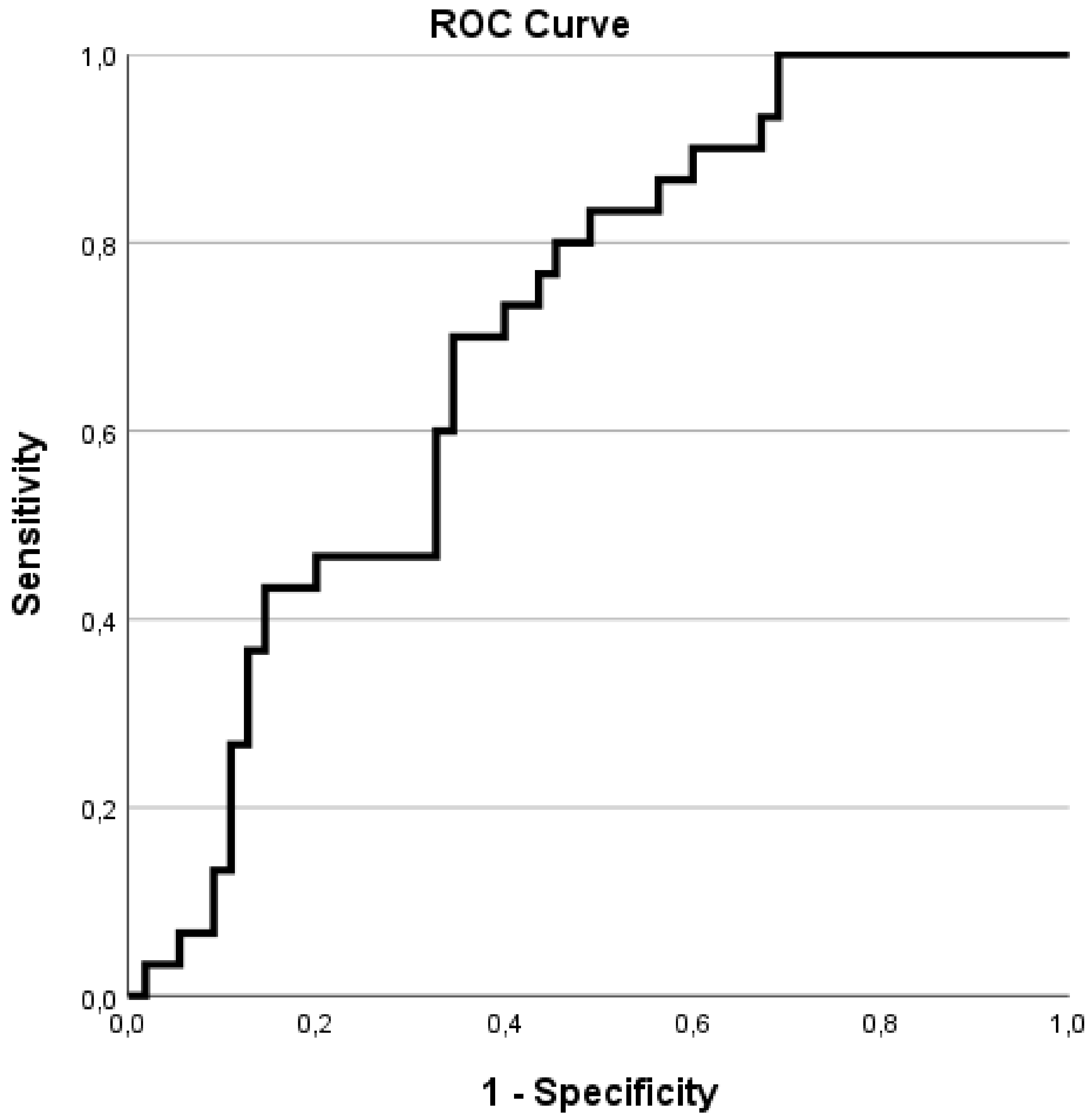

**AUC**	0.70	95% CI 0.54–0.81
**Optimal cut-off**	-0.14	
**Sensitivity**	80%	
**Specificity**	55%	
