# Supplementary material for: Exploration of circulating proteins associated with tumor stromal content using matched serum and tissue samples from patients with colon cancer
Source: J Liq Biopsy. 2026 Oct 23;14:100688. doi: 10.1016/j.jlb.2026.100688 (PMC13734802; doi:10.1016/j.jlb.2026.100688)

Kallistatin

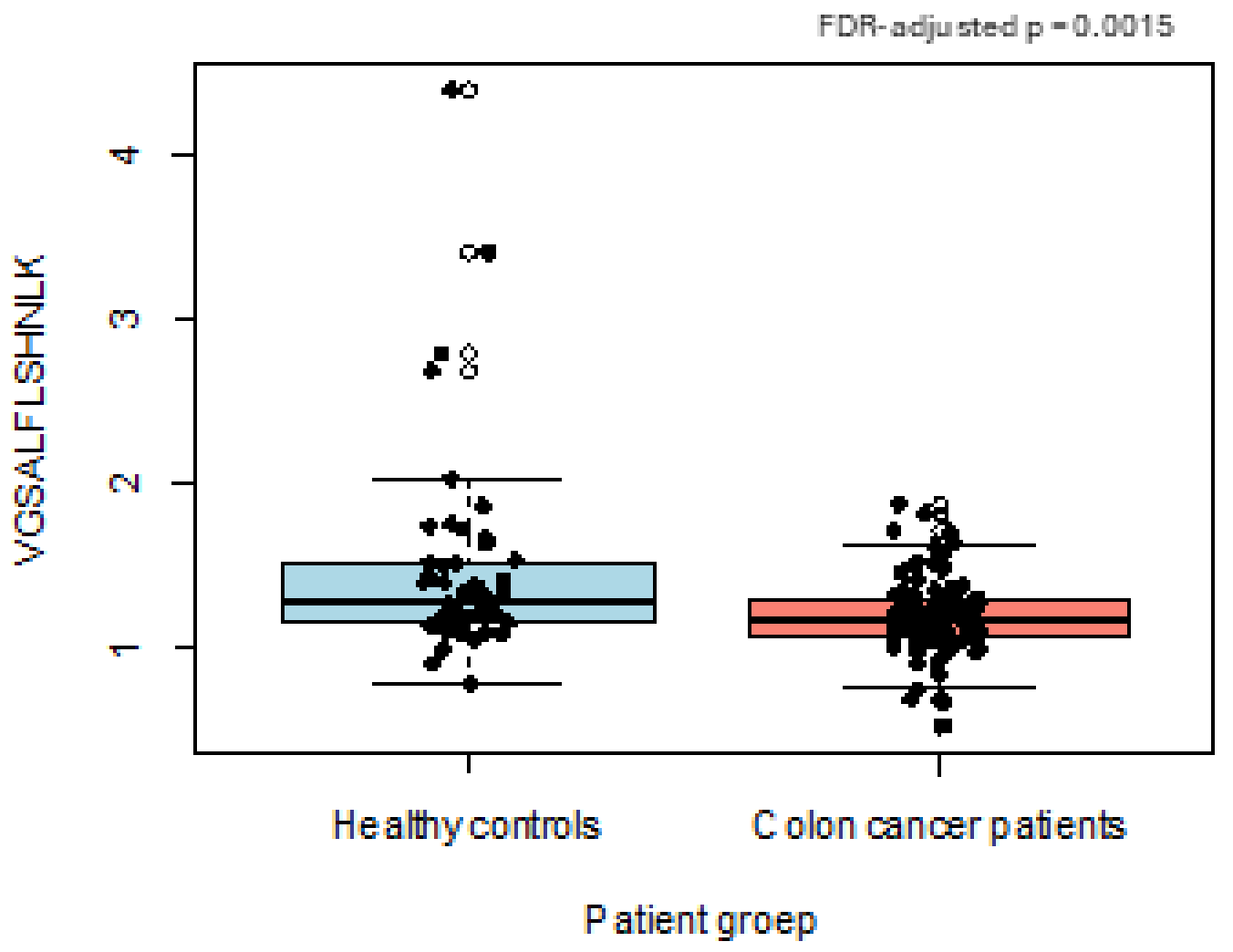

Actin, aortic smooth muscle

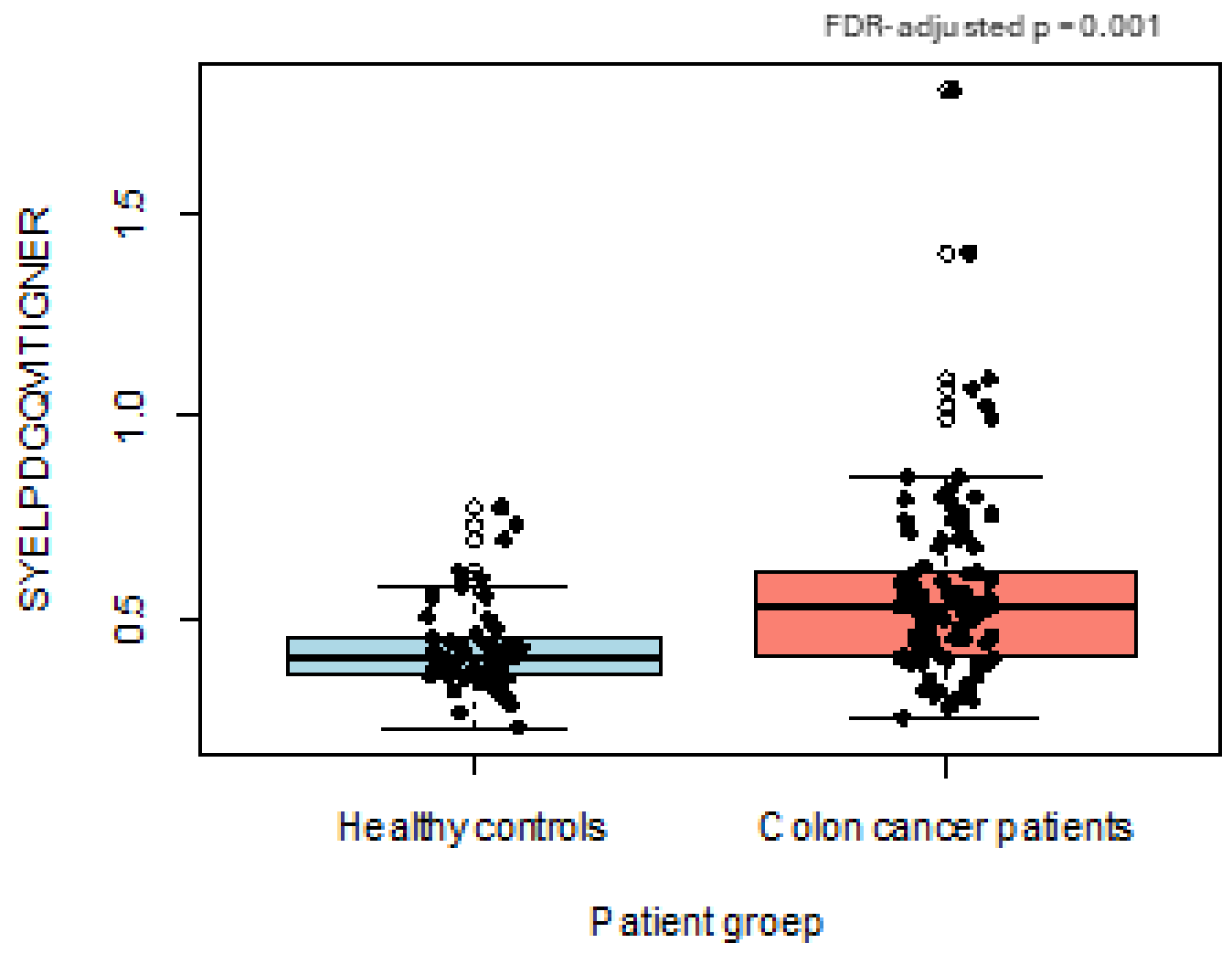

Cholinesterase

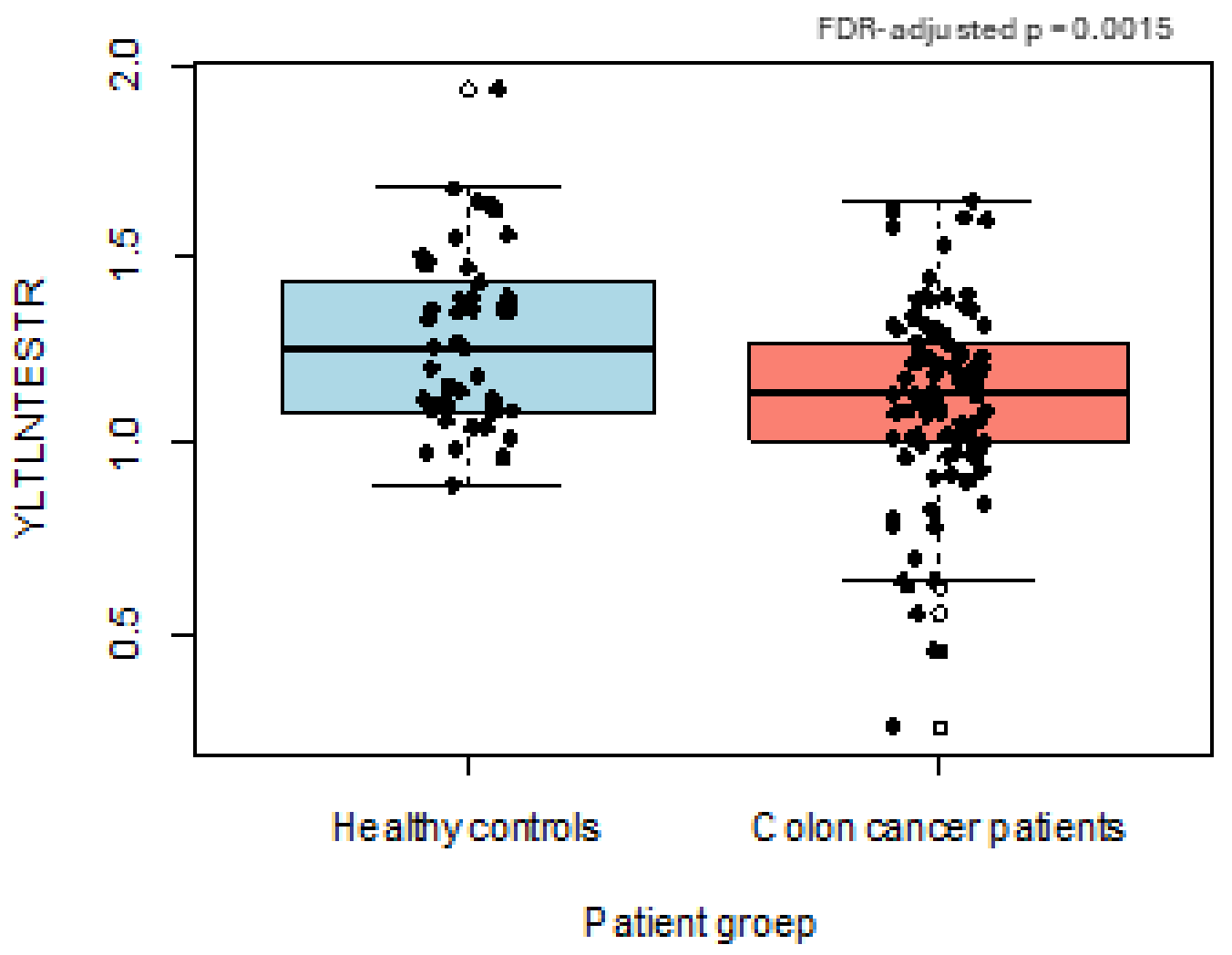

Transthyretin

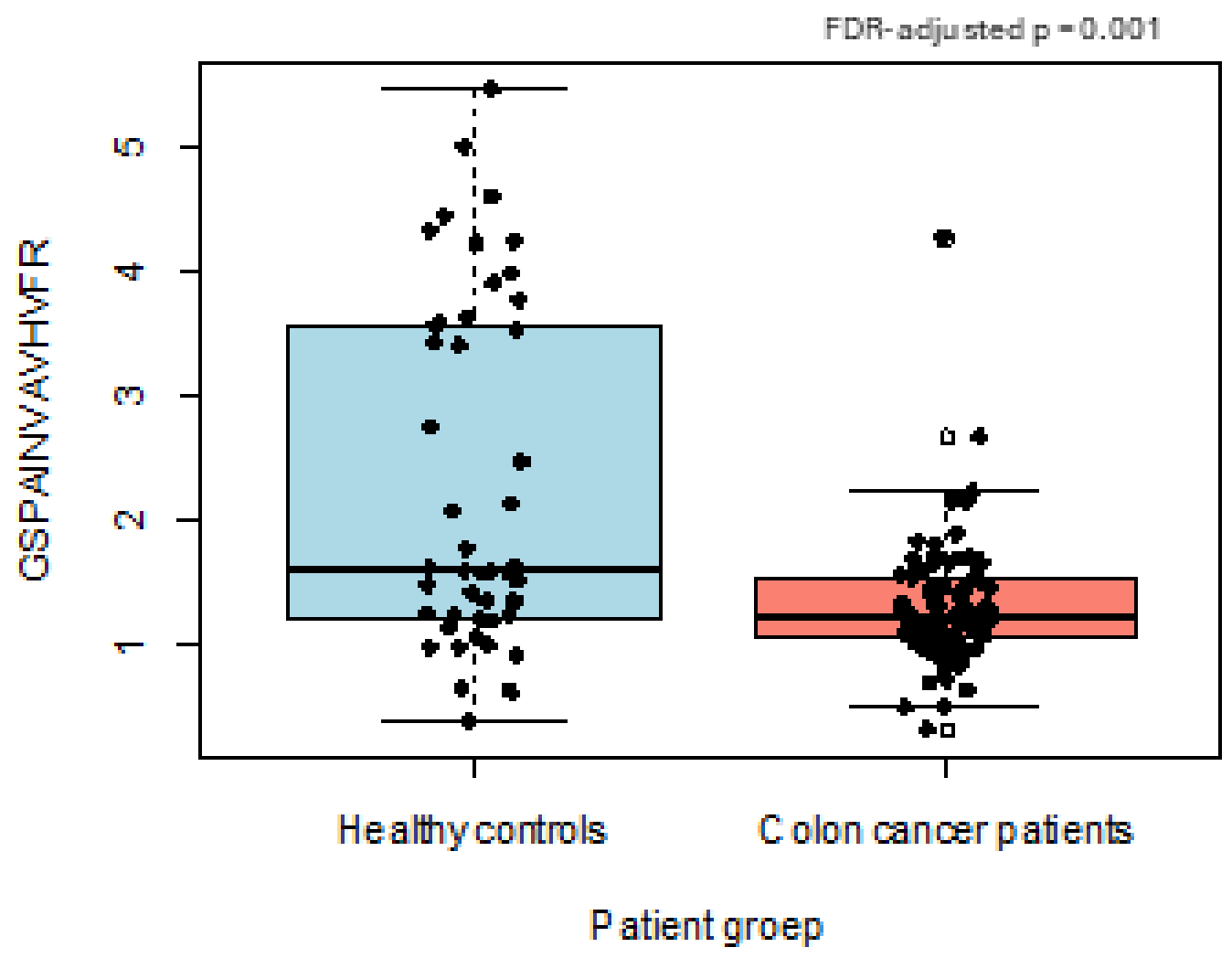

Thrombospondin-1

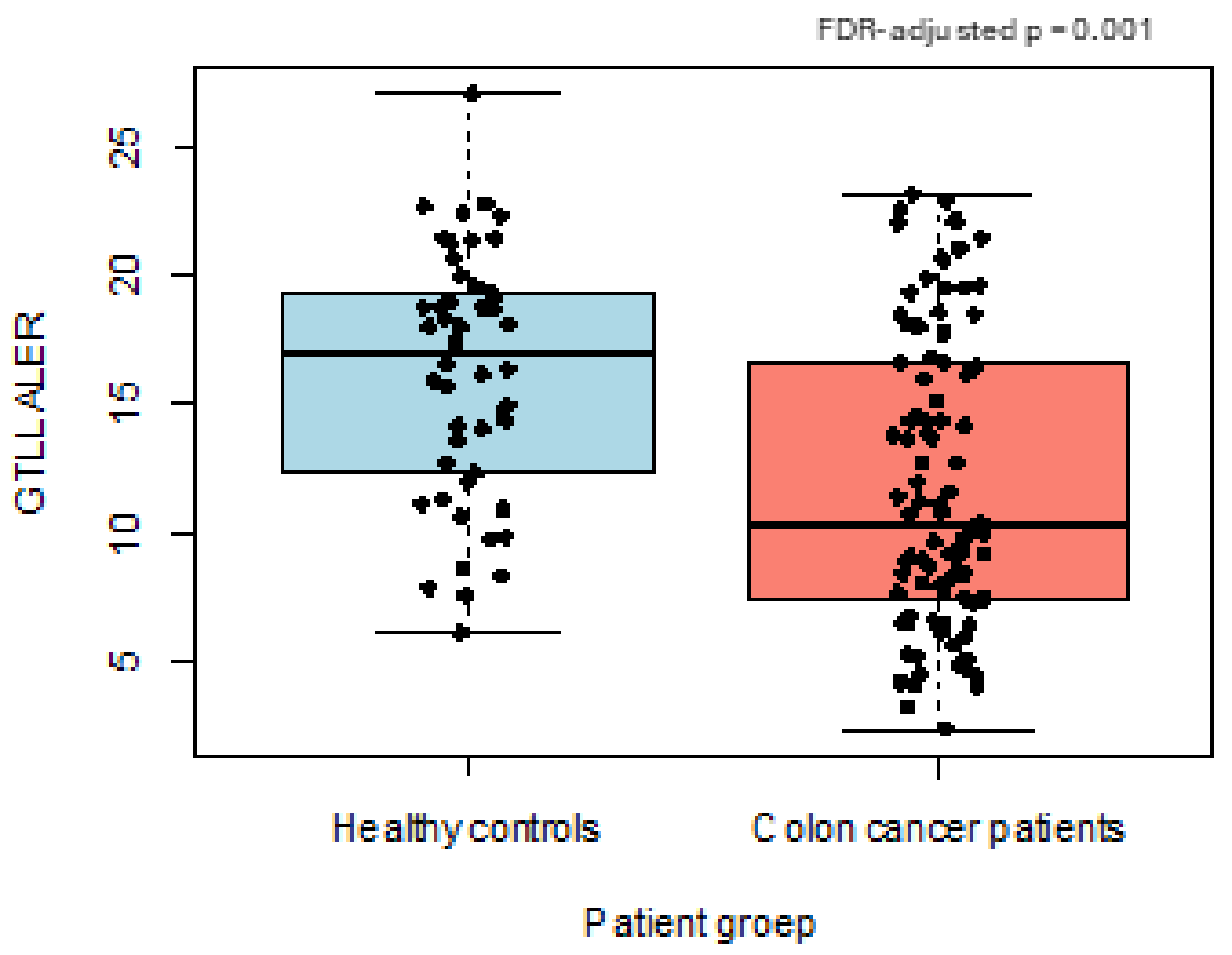

Extracellular matrix protein 1

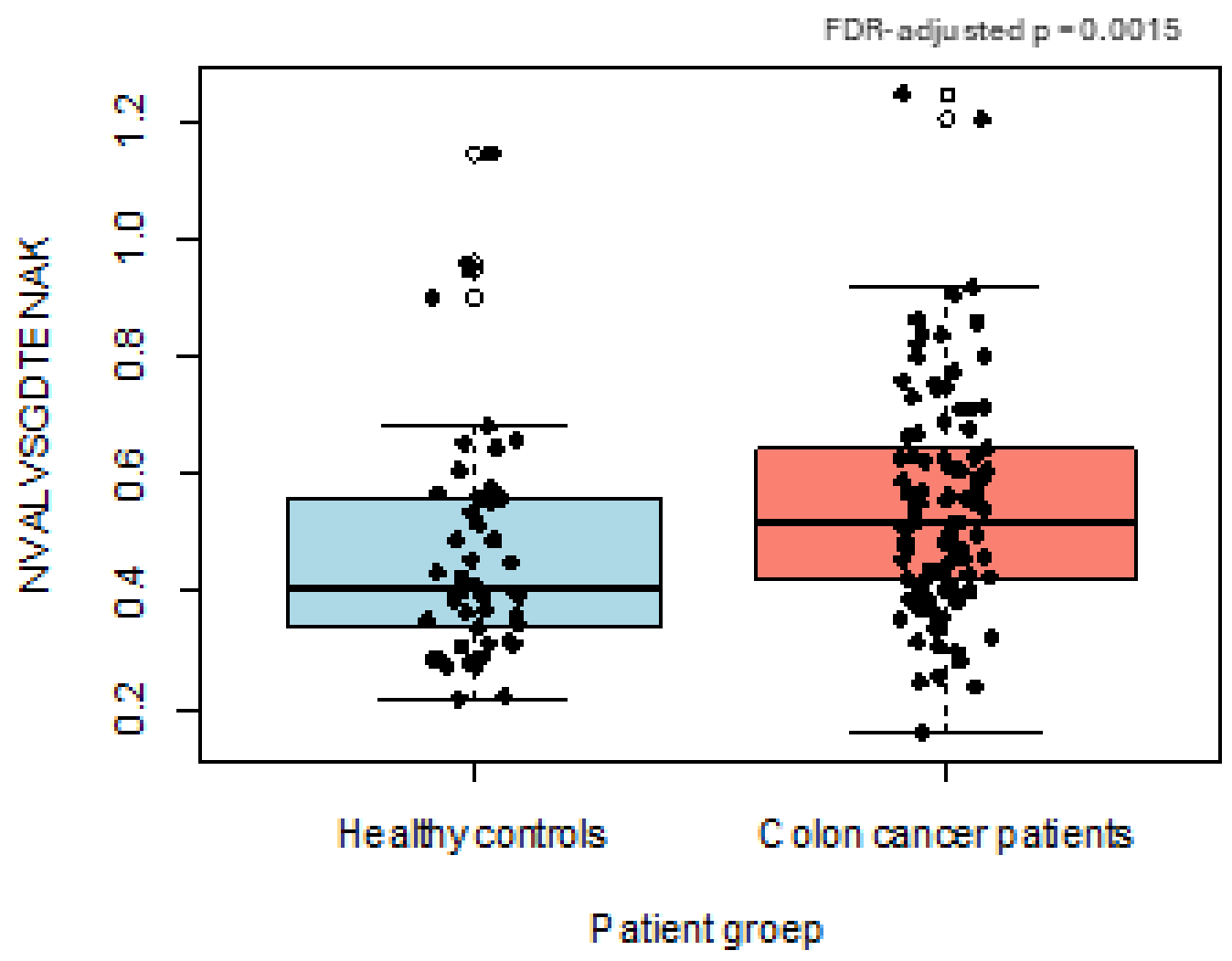

Supplement: Supplementary file 4 [file mmc4.pdf]
