# Supplementary material for: Exploration of circulating proteins associated with tumor stromal content using matched serum and tissue samples from patients with colon cancer
Source: J Liq Biopsy. 2026 Oct 23;14:100688. doi: 10.1016/j.jlb.2026.100688 (PMC13734802; doi:10.1016/j.jlb.2026.100688)
Supplement: Supplementary file 5 [file mmc5.docx]

**Supplementary Table 1.** Overview of proteins with corresponding peptides and their secretome function as annotated by the Human Protein atlas.

| **Protein** | **Peptide** | **UniProtKB Accession Number** | **Protein abbreviation** | **Secretome function** |
| --- | --- | --- | --- | --- |
| Actin. aortic smooth muscle | NWGLSVYADKPETTK | P02763 | AGP1 | Acute phase |
| Adhesion G protein-coupled receptor F5 | EIGELYLPK | P01011 | ACT | Acute phase |
| Adipocyte plasma membrane-associated protein | LGNQEPGGQTALK | P08697-1 | A2AP | Acute phase |
| Adiponectin | AFVFPK | P02741 | CRP | Acute phase |
| Afamin | HTSVQTTSSGSGPFTDVR | P02751 | FN | Acute phase |
| Alpha-1-acid glycoprotein 1 | DIAPTLTLYVGK | P00738 | Hp | Acute phase |
| Alpha-1-antichymotrypsin | SPEQQETVLDGNLIIR | Q14624 | ITI-HC4 | Acute phase |
| Alpha-1-antitrypsin | EANYIGSDK | P0DJI8 | SAA1 | Acute phase |
| Alpha-1B-glycoprotein VAR 018369 | GNYDAAQR | P35542 | SAA4 | Acute phase |
| Alpha-2-antiplasmin | ATEHLSTLSEK | P02647 | ApoA-I | Apolipoprotein |
| Alpha-2-HS-glycoprotein | SPELQAEAK | P02652 | ApoA-II | Apolipoprotein |
| Alpha-2-macroglobulin | LGEVNTYAGDLQK | P06727 | ApoA-IV | Apolipoprotein |
| Angiotensinogen | FPEVDVLTK | P04114 | ApoB-100 | Apolipoprotein |
| Antithrombin-III | EWFSETFQK | P02654 | ApoC-I | Apolipoprotein |
| Apolipoprotein A-I | TYLPAVDEK | P02655 | ApoC-II | Apolipoprotein |
| Apolipoprotein A-II | GWVTDGFSSLK | P02656 | ApoC-III | Apolipoprotein |
| Apolipoprotein A-IV | ELLETVVNR | P55056 | ApoC-IV | Apolipoprotein |
| Apolipoprotein B-100 | NILTSNNIDVK | P05090 | ApoD | Apolipoprotein |
| Apolipoprotein C-I | LGPLVEQGR | P02649 | ApoE | Apolipoprotein |
| Apolipoprotein C-II | SGVQQLIQYYQDQK | Q13790 | ApoF | Apolipoprotein |
| Apolipoprotein C-III | VAQELEEK | O14791 | ApoL1 | Apolipoprotein |
| Apolipoprotein C-IV | AFLLTPR | O95445 | ApoM | Apolipoprotein |
| Apolipoprotein D | ATVVYQGER | P02749 | β2GP1 | Apolipoprotein |
| Apolipoprotein E | SVLGQLGITK | P01009 | AAT | Blood coagulation |
| Apolipoprotein F | DDLYVSDAFHK | P01008 | ATIII | Blood coagulation |
| Apolipoprotein L1 | SALVLQYLR | P00740 | FIX | Blood coagulation |
| Apolipoprotein M | MLEVPYVDR | P00742 | FX | Blood coagulation |
| Apolipoprotein(a) | TSESGLPSTR | P03951 | FXI | Blood coagulation |
| Attractin | EQPPSLTR | P00748 | FXII | Blood coagulation |
| Beta-2-glycoprotein 1 | GTYIPVPIVSELQSGK | P00488 | FXIIIA | Blood coagulation |
| Beta-2-microglobulin | IQTHSTTYR | P05160 | FXIIIB | Blood coagulation |
| Beta-Ala-His dipeptidase | VQHIQLLQK | P02671 | FGA | Blood coagulation |
| Biotinidase | HQLYIDETVNSNIPTNLR | P02675 | FGB | Blood coagulation |
| C-reactive protein | YEASILTHDSSIR | P02679 | FGG | Blood coagulation |
| C4b-binding protein alpha chain | TLEAQLTPR | P05546 | HLS2 | Blood coagulation |
| Cadherin-5 | ADLFYDVEALDLESPK | P04196 | HPRG | Blood coagulation |
| Carbonic anhydrase 1 | FQPTLLTLPR | P05155 | C1-Inh | Blood coagulation |
| Carboxypeptidase B2 | EAQLPVIENK | P00747 | PLG | Blood coagulation |
| Carboxypeptidase N catalytic chain | ELLESYIDGR | P00734 | FII | Blood coagulation |
| Carboxypeptidase N subunit 2 | IAWHVIR | Q96IY4 | TAFI | Blood coagulation |
| Cartilage acidic protein 1 | LGEYDLR | P04070 | PROC | Blood coagulation |
| Cathelicidin antimicrobial peptide | VYFAGFPR | P07225 | PROS | Blood coagulation |
| CD5 antigen-like | DFAEHLLIPR | P22891 | PROZ | Blood coagulation |
| cDNA FLJ53327 | LAELPADALGPLQR | P35858 | ALS | Cell adhesion |
| Ceruloplasmin | GTLLALER | P07996 | TSP1 | Cell adhesion |
| Cholinesterase | NTVISVNPSTK | P19320 | VCAM-1 | Cell adhesion |
| Clusterin | FEDGVLDPDYPR | P04004 | VN | Cell adhesion |
| Coagulation factor IX | EDVYVVGTVLR | P04003 | C4bp | Complement pathway |
| Coagulation factor X | ELDESLQVAER | P10909 | CLU | Complement pathway |
| Coagulation factor XI | PAFSAIR | P02745 | C1QA | Complement pathway |
| Coagulation factor XII | IAFSATR | P02746 | C1QB | Complement pathway |
| Coagulation factor XIII A chain | FQSVFTVTR | P02747 | C1QC | Complement pathway |
| Coagulation factor XIII B chain | GLTLHLK | P00736 | C1R | Complement pathway |
| Complement C1q subcomponent subunit A | VVVHPDYR | Q9NZP8 | C1RL | Complement pathway |
| Complement C1q subcomponent subunit B | TNFDNDIALVR | P09871 | C1S | Complement pathway |
| Complement C1q subcomponent subunit C | HAFILQDTK | P06681 | C2 | Complement pathway |
| Complement C1r subcomponent-like protein | TGLQEVEVK | P01024 | C3 | Complement pathway |
| Complement C1r subcomponent | VGDTLNLNLR | P0C0L4 | C4A | Complement pathway |
| Complement C1s subcomponent | VFQFLEK | P01031 | C5 | Complement pathway |
| Complement C2 | DLHLSDVFLK | P13671 | C6 | Complement pathway |
| Complement C3 | AASGTQNNVLR | P10643 | C7 | Complement pathway |
| Complement C4 | MESLGITSR | P07357 | C8A | Complement pathway |
| Complement C5 | SDLEVAHYK | P07358 | C8B | Complement pathway |
| Complement component C6 | LSPIYNLVPVK | P02748 | C9 | Complement pathway |
| Complement component C7 | VFSLQWGEVK | P05156 | CFI | Complement pathway |
| Complement component C8 alpha chain | NVALVSGDTENAK | Q16610 | ECM1 | Developmental protein |
| Complement component C8 beta chain | ALEQDLPVNIK | Q96KN2 | CNDP1 | Enzyme |
| Complement component C9 | SHLIIAQVAK | P43251 | BTD | Enzyme |
| Complement factor B | SIPQVSPVR | P15169 | CPN | Enzyme |
| Complement factor D | IYHSHIDAPK | P00450 | CP | Enzyme |
| Complement factor H | YLTLNTESTR | P06276 | BChE | Enzyme |
| Complement factor I | QEPGENSEILPTLK | P22352 | GPx-3 | Enzyme |
| Corticosteroid-binding globulin | VVLGDQDLK | Q14520 | HABP2 | Enzyme |
| Creatine kinase M-type | AWVAWR | P61626 | LYZ | Enzyme |
| Cystatin-C | SSGLVSNAPGVQIR | P04180 | LCAT | Enzyme |
| Extracellular matrix protein 1 | FGSSLITVR | P80108-1 | Gpld1 | Enzyme |
| Fetuin-B | HHGPTITAK | P02760 | AMBP | Enzyme |
| Fibrinogen alpha chain | IFFYDSENPPASEVLR | P27169 | PON1 | Enzyme |
| Fibrinogen beta chain | ILIGTVFHK | Q15166 | PON3 | Enzyme |
| Fibrinogen gamma chain | AIGYLNTGYQR | P01023 | A2M | Enzyme inhibitor |
| Fibronectin | ALDFAVGEYNK | P01034 | CysC | Enzyme inhibitor |
| Fibulin-1 | LVVLPFPK | Q9UGM5 | FETUB | Enzyme inhibitor |
| Ficolin-3 | GSLVQASEANLQAAQDFVR | P19827 | ITI-HC1 | Enzyme inhibitor |
| Follistatin-related protein 1 | SLAPTAAAK | P19823 | ITI-HC2 | Enzyme inhibitor |
| Gelsolin | VGSALFLSHNLK | P29622 | KAL | Enzyme inhibitor |
| Glutathione peroxidase 3 | EYLIAGK | P16035 | TIMP-2 | Enzyme inhibitor |
| Haptoglobin | ISEITNIVSK | P20742 | PZP | Enzyme inhibitor |
| Hemoglobin subunit alpha | DLIQHPK | O43915 | VEGF-D | Growth factor |
| Hemopexin | IFYNQQNHYDGSTGK | Q15848 | AdipoQ | Hormone |
| Heparin cofactor 2 | GSPAINVAVHVFR | P02766 | ATTR | Hormone |
| Histidine-rich glycoprotein | VNHVTLSQPK | P61769 | B2M | Immunity |
| Hyaluronan-binding protein 2 | AIDGINQR | P49913 | CAP-18 | Immunity |
| Ig gamma-1 chain C region | LVGGLHR | O43866 | CD5L | Immunity |
| Ig mu chain C region | EELLPAQDIK | P00751-1 | CFB | Immunity |
| Ig mu heavy chain disease protein and Ig mu chain C | THHDGAITER | P00746 | CFD | Immunity |
| IgGFc-binding protein | SSQESYAHGTK | P08603 | CFH | Immunity |
| Immunoglobulin kappa variable 4-1 | YAVSEAAAHK | O75636 | FCN3 | Immunity |
| Insulin-like growth factor-binding protein 2 | GPSVFPLAPSSK | P01857 | IGHG1 | Immunity |
| Insulin-like growth factor-binding protein 3 | GFPSVLR | P01871 | IGHM | Immunity |
| Insulin-like growth factor-binding protein complex acid labile subunit | VSVFVPPR | P04220 | IGHM | Immunity |
| Inter-alpha-trypsin inhibitor heavy chain H1 | NYLAWYQQKPGQPPK | P06312 | IGKV4-1 | Immunity |
| Inter-alpha-trypsin inhibitor heavy chain H2 | TGVITSPDFPNPYPK | P48740 | MASP1 | Immunity |
| Inter-alpha-trypsin inhibitor heavy chain H4 | AGLLRPDYALLGHR | Q96PD5 | NAMLAA | Immunity |
| Kallistatin | DLQNFLK | P06702 | S1-A9 | Immunity |
| Kininogen-1 | SYELPDGQVITIGNER | P62736 | ACTA2 | No annotated function |
| L-selectin | DVIVHPLPLK | Q8IZF2 | GPR116 | No annotated function |
| Leucine-rich alpha-2-glycoprotein | LLEYDTVTR | Q9HDC9 | APMAP | No annotated function |
| Lipopolysaccharide-binding protein | LETPDFQLFK | P04217-1 | A1BG | No annotated function |
| Lumican | ELDSTGTPTGK | P33151 | CDH5 | No annotated function |
| Lysozyme C | VNVGVK | P51911 | CNN1 | No annotated function |
| Mannan-binding lectin serine protease 1 | VLDALQAIK | P00915 | CA-I | No annotated function |
| Mannose-binding protein C | AGGSWDLAVQER | P22792 | CPN2 | No annotated function |
| Melanotransferrin | GVASLFAGR | Q9NQ79 | CRTAC1 | No annotated function |
| Metalloproteinase inhibitor 2 | EGGQTAPASTR | B7Z2X4 | cDNA FLJ53327 | No annotated function |
| N-acetylmuramoyl-L-alanine amidase | VGAHAGEYGAEALER | P69905 | HBA1 | No annotated function |
| Neuropilin-2 | GATTSPGVYELSSR | Q9Y6R7 | FCGBP | No annotated function |
| Neutrophil gelatinase-associated lipocalin | AEIEYLEK | P14151 | CD62L | No annotated function |
| Peroxiredoxin-2 | DLLLPQPDLR | P02750 | LRG | No annotated function |
| Phosphatidylcholine-sterol acyltransferase | SLEDLQLTHNK | P51884 | LUM | No annotated function |
| Phosphatidylinositol-glycan-specific phospholipase D | WLTFSLGK | P11226 | MBP-C | No annotated function |
| Phospholipid transfer protein | ELGPYTLDR | Q8WXI7 | MUC-16 | No annotated function |
| Pigment epithelium-derived factor | GLFIIDGK | P32119-1 | PRDX2 | No annotated function |
| Plasma protease C1 inhibitor | LQSLFDSPDFSK | P36955 | PEDF | No annotated function |
| Plasma serine protease inhibitor | ALVILAK | Q99497 | PARK7 | No annotated function |
| Plasminogen | DQYYNIDVPSR | Q92954 | PRG4 | No annotated function |
| Pregnancy zone protein | IVLGQEQDSYGGK | P02743 | SAP | No annotated function |
| Protein AMBP | LEAGDHPVELLAR | P09486 | SPARC | No annotated function |
| Protein deglycase DJ-1 | IYLSDSLTGK | Q7Z7G0 | Tarsh | No annotated function |
| Protein S100-A9 | NWETEITAQPDGGK | P05452 | TN | No annotated function |
| Proteoglycan 4 | YLQGSSVQLR | Q6EMK4 | Vasn | No annotated function |
| Prothrombin | AVYEAVLR | P12955 | XPD | No annotated function |
| Retinol-binding protein 4 | EIPAWVPFDPAAQITK | P25311 | Zn-alpha-2-GP | No annotated function |
| Serotransferrin | FSVVYAK | P02765 | AHSG | Other |
| Serum albumin | ALQDQLVLVAAK | P01019 | Ang-I | Other |
| Serum amyloid A-1 and A-2 proteins | TGYYFDGISR | P23142 | FIBL-1 | Other |
| Serum amyloid A-4 protein | YVQELQK | Q12841 | FSTL1 | Other |
| Serum amyloid P-component | AGALNSNDAFVLK | P06396 | GSN | Other |
| Serum paraoxonase/arylesterase 1 | LIQGAPTIR | P18065 | IGFBP-2 | Other |
| Serum paraoxonase/lactonase 3 | FLNVLSPR | P17936 | IGFBP-3 | Other |
| Sex hormone-binding globulin | SVNNVVVR | O75882 | ATRN | Receptor |
| SPARC | ALQVVR | O60462 | NRP2 | Receptor |
| Target of Nesh-SH3 | EATLELLGR | P19438 | TNF-R1 | Receptor |
| Tetranectin | DADPDTFFAK | P43652 | Alpha-Alb | Transport |
| Thrombospondin-1 | GTYSTTVTGR | P08519 | Apo(a) | Transport |
| Thyroxine-binding globulin | WSAGLTSSQVDLYIPK | P08185 | CBG | Transport |
| Transthyretin | NFPSPVDAAFR | P02790 | Hpx | Transport |
| Vascular cell adhesion protein 1 | YYDYSGAFR | P08582 | CD228 | Transport |
| Vascular endothelial growth factor D | ITLYGR | P80188 | NGAL | Transport |
| Vasorin | AVEPQLQEEER | P55058 | PLTP | Transport |
| Vitamin D-binding protein | GFQQLLQELNQPR | P05154 | PAI-3 | Transport |
| Vitamin K-dependent protein C | YWGVASFLQK | P02753 | RBP4 | Transport |
| Vitamin K-dependent protein S | DGAGDVAFVK | P02787 | Tf | Transport |
| Vitamin K-dependent protein Z | LVNEVTEFAK | P02768 | ALB | Transport |
| Vitronectin | TSSSFEVR | P04278 | SHBG | Transport |
| Xaa-Pro dipeptidase | AVLHIGEK | P05543 | TBG | Transport |
| Zinc-alpha-2-glycoprotein | VLEPTLK | P02774 | DBP | Transport |
