# Supplementary material for: Exploration of circulating proteins associated with tumor stromal content using matched serum and tissue samples from patients with colon cancer
Source: J Liq Biopsy. 2026 Oct 23;14:100688. doi: 10.1016/j.jlb.2026.100688 (PMC13734802; doi:10.1016/j.jlb.2026.100688)
Supplement: Supplementary file 6 [file mmc6.docx]

**Supplementary Table 2.** Variation in quality control samples in validation- and sample runs.

| **Protein** | **Quality Control Serum Pools** | |
| --- | --- | --- |
|  | **QC1** | **QC2** |
| Actin. aortic smooth muscle | 2.3% | 2.2% |
| Adhesion G protein-coupled receptor F5 | 9.4% | 3.7% |
| Adipocyte plasma membrane-associated protein | 6.0% | 3.5% |
| Adiponectin | 10.6% | 9.8% |
| Afamin | 7.2% | 9.7% |
| Alpha-1-acid glycoprotein 1 | 8.4% | 8.6% |
| Alpha-1-antichymotrypsin | 3.0% | 4.1% |
| Alpha-1-antitrypsin | 8.1% | 9.3% |
| Alpha-1B-glycoprotein VAR 018369 | 5.9% | 3.2% |
| Alpha-2-antiplasmin | 2.4% | 1.8% |
| Alpha-2-HS-glycoprotein | 2.5% | 2.1% |
| Alpha-2-macroglobulin | 2.5% | 1.5% |
| Angiotensinogen | 1.2% | 1.0% |
| Antithrombin-III | 8.5% | 6.2% |
| Apolipoprotein A-I | 5.6% | 3.0% |
| Apolipoprotein A-II | 3.8% | 2.6% |
| Apolipoprotein A-IV | 2.7% | 2.5% |
| Apolipoprotein B-100 | 3.3% | 1.8% |
| Apolipoprotein C-I | 9.7% | 10.0% |
| Apolipoprotein C-II | 4.6% | 2.8% |
| Apolipoprotein C-III | 3.2% | 1.6% |
| Apolipoprotein C-IV | 4.4% | 3.1% |
| Apolipoprotein D | 4.1% | 2.4% |
| Apolipoprotein E | 3.4% | 3.4% |
| Apolipoprotein F | 7.3% | 8.6% |
| Apolipoprotein L1 | 1.8% | 2.4% |
| Apolipoprotein M | 3.3% | 1.6% |
| Apolipoprotein(a) | 6.8% | 4.3% |
| Attractin | 2.6% | 2.1% |
| Beta-2-glycoprotein 1 | 2.8% | 3.0% |
| Beta-2-microglobulin | 7.4% | 4.6% |
| Beta-Ala-His dipeptidase | 3.6% | 4.9% |
| Biotinidase | 11.4% | 5.5% |
| C-reactive protein | 2.9% | 2.0% |
| C4b-binding protein alpha chain | 3.6% | 3.7% |
| Cadherin-5 | 14.9% | 8.4% |
| Carbonic anhydrase 1 | 7.2% | 6.0% |
| Carboxypeptidase B2 | 5.5% | 2.5% |
| Carboxypeptidase N catalytic chain | 3.8% | 2.3% |
| Carboxypeptidase N subunit 2 | 7.4% | 4.5% |
| Cartilage acidic protein 1 | 4.3% | 6.7% |
| Cathelicidin antimicrobial peptide | 8.3% | 3.7% |
| CD5 antigen-like | 6.2% | 3.7% |
| cDNA FLJ53327 | 6.6% | 1.9% |
| Ceruloplasmin | 1.8% | 1.4% |
| Cholinesterase | 5.4% | 3.3% |
| Clusterin | 2.4% | 4.0% |
| Coagulation factor IX | 6.5% | 8.4% |
| Coagulation factor X | 5.6% | 7.5% |
| Coagulation factor XI | 19.3% | 19.0% |
| Coagulation factor XII | 2.8% | 1.0% |
| Coagulation factor XIII A chain | 21.7% | 21.6% |
| Coagulation factor XIII B chain | 3.1% | 3.7% |
| Complement C1q subcomponent subunit A | 4.9% | 3.7% |
| Complement C1q subcomponent subunit B | 3.4% | 1.6% |
| Complement C1q subcomponent subunit C | 4.2% | 3.7% |
| Complement C1r subcomponent-like protein | 4.5% | 5.0% |
| Complement C1r subcomponent | 0.8% | 3.7% |
| Complement C1s subcomponent | 1.0% | 2.7% |
| Complement C2 | 12.8% | 9.8% |
| Complement C3 | 2.4% | 1.7% |
| Complement C4 | 1.8% | 1.5% |
| Complement C5 | 2.9% | 3.7% |
| Complement component C6 | 66.9% | 40.7% |
| Complement component C7 | 3.2% | 2.6% |
| Complement component C8 alpha chain | 3.2% | 1.5% |
| Complement component C8 beta chain | 2.9% | 2.7% |
| Complement component C9 | 3.7% | 4.5% |
| Complement factor B | 5.2% | 3.0% |
| Complement factor D | 7.9% | 5.8% |
| Complement factor H | 5.0% | 3.1% |
| Complement factor I | 4.2% | 2.1% |
| Corticosteroid-binding globulin | 6.6% | 7.1% |
| Creatine kinase M-type | 15.0% | 9.0% |
| Cystatin-C | 12.5% | 4.4% |
| Extracellular matrix protein 1 | 7.3% | 4.3% |
| Fetuin-B | 1.9% | 2.1% |
| Fibrinogen alpha chain | N.D. | N.D. |
| Fibrinogen beta chain | N.D. | N.D. |
| Fibrinogen gamma chain | N.D. | N.D. |
| Fibronectin | 4.8% | 3.2% |
| Fibulin-1 | 4.5% | 2.5% |
| Ficolin-3 | 6.4% | 4.4% |
| Follistatin-related protein 1 | 14.6% | 14.7% |
| Gelsolin | 3.1% | 3.2% |
| Glutathione peroxidase 3 | 3.1% | 6.3% |
| Haptoglobin | 9.8% | 7.9% |
| Hemoglobin subunit alpha | 8.1% | 6.9% |
| Hemopexin | 3.1% | 1.6% |
| Heparin cofactor 2 | 2.8% | 1.3% |
| Histidine-rich glycoprotein | 2.6% | 1.3% |
| Hyaluronan-binding protein 2 | 4.6% | 2.5% |
| Ig gamma-1 chain C region | 2.8% | 3.6% |
| Ig mu chain C region | 2.5% | 3.2% |
| Ig mu heavy chain disease protein and Ig mu chain C | 2.8% | 1.7% |
| IgGFc-binding protein | 16.3% | 10.5% |
| Immunoglobulin kappa variable 4-1 | 4.1% | 9.4% |
| Insulin-like growth factor-binding protein 2 | 8.1% | 8.9% |
| Insulin-like growth factor-binding protein 3 | 3.3% | 3.2% |
| Insulin-like growth factor-binding protein complex acid labile subunit | 1.7% | 1.3% |
| Inter-alpha-trypsin inhibitor heavy chain H1 | 5.5% | 5.2% |
| Inter-alpha-trypsin inhibitor heavy chain H2 | 2.4% | 1.5% |
| Inter-alpha-trypsin inhibitor heavy chain H4 | 4.0% | 2.0% |
| Kallistatin | 7.8% | 18.8% |
| Kininogen-1 | 2.5% | 1.6% |
| L-selectin | 4.7% | 7.0% |
| Leucine-rich alpha-2-glycoprotein | 1.6% | 1.6% |
| Lipopolysaccharide-binding protein | 2.2% | 6.5% |
| Lumican | 2.7% | 2.3% |
| Lysozyme C | 11.2% | 34.4% |
| Mannan-binding lectin serine protease 1 | 6.2% | 2.6% |
| Mannose-binding protein C | 10.4% | 10.6% |
| Melanotransferrin | 33.4% | 124.2% |
| Metalloproteinase inhibitor 2 | 7.0% | 9.2% |
| N-acetylmuramoyl-L-alanine amidase | 8.4% | 6.5% |
| Neuropilin-2 | 3.0% | 2.0% |
| Neutrophil gelatinase-associated lipocalin | 6.4% | 8.9% |
| Peroxiredoxin-2 | 2.7% | 2.6% |
| Phosphatidylcholine-sterol acyltransferase | 4.5% | 2.9% |
| Phosphatidylinositol-glycan-specific phospholipase D | 2.5% | 3.1% |
| Phospholipid transfer protein | 3.2% | 3.4% |
| Pigment epithelium-derived factor | 2.1% | 2.9% |
| Plasma protease C1 inhibitor | 1.5% | 2.5% |
| Plasma serine protease inhibitor | 39.3% | 13.6% |
| Plasminogen | 3.4% | 1.9% |
| Pregnancy zone protein | 4.1% | 18.9% |
| Protein AMBP | 1.9% | 1.7% |
| Protein deglycase DJ-1 | 23.6% | 13.8% |
| Protein S100-A9 | 23.6% | 29.5% |
| Proteoglycan 4 | 15.0% | 14.1% |
| Prothrombin | 2.8% | 1.0% |
| Retinol-binding protein 4 | 32.7% | 19.5% |
| Serotransferrin | 2.8% | 4.6% |
| Serum albumin | 3.1% | 1.2% |
| Serum amyloid A-1 and A-2 proteins | 3.9% | 7.0% |
| Serum amyloid A-4 protein | 3.1% | 1.5% |
| Serum amyloid P-component | 9.2% | 8.8% |
| Serum paraoxonase/arylesterase 1 | 2.4% | 2.3% |
| Serum paraoxonase/lactonase 3 | 2.6% | 2.8% |
| Sex hormone-binding globulin | 6.1% | 5.7% |
| SPARC | 11.0% | 10.9% |
| Target of Nesh-SH3 | 19.9% | 66.3% |
| Tetranectin | 5.4% | 8.9% |
| Thrombospondin-1 | 4.2% | 4.4% |
| Thyroxine-binding globulin | 2.5% | 3.6% |
| Transthyretin | 22.3% | 22.5% |
| Vascular cell adhesion protein 1 | 11.6% | 10.2% |
| Vascular endothelial growth factor D | 54.3% | 92.3% |
| Vasorin | 9.7% | 9.8% |
| Vitamin D-binding protein | 2.4% | 1.4% |
| Vitamin K-dependent protein C | 6.6% | 6.3% |
| Vitamin K-dependent protein S | 3.3% | 2.5% |
| Vitamin K-dependent protein Z | 7.0% | 4.1% |
| Vitronectin | 3.2% | 2.3% |
| Xaa-Pro dipeptidase | 2.4% | 9.9% |
| Zinc-alpha-2-glycoprotein | 13.3% | 15.5% |

Across five independent runs of citrate quality control (QC) samples, coefficients of variation were below 15% for 146 proteins and below 7% for 123 proteins.
