# Supplementary material for: Exploration of circulating proteins associated with tumor stromal content using matched serum and tissue samples from patients with colon cancer
Source: J Liq Biopsy. 2026 Oct 23;14:100688. doi: 10.1016/j.jlb.2026.100688 (PMC13734802; doi:10.1016/j.jlb.2026.100688)
Supplement: Supplementary file 7 [file mmc7.docx]

**Supplementary Table 3A**. Multivariable analysis including TSR demonstrating no independent prognostic value of Stromal Protein Score for disease-free survival.

| **Variable** | **HR** | **95% CI** | **p-value** |
| --- | --- | --- | --- |
| *Stromal Protein Score* | | | |
| Male sex | 0.593 | 0.179 – 1.966 | 0.393 |
| Right-sided colon cancer | 3.193 | 0.988 – 10.314 | 0.052 |
| Stroma-high tumor | 0.470 | 0.113 – 1.960 | 0.300 |
| Tumor Stage | - | - | 0.213 |
| ACT given | 1.977 | 0.348 – 11.238 | 0.442 |
| Stromal Protein Score | 1.856 | 0.677 – 5.086 | 0.229 |

TSR = Tumor-Stroma Ratio, HR = hazard ratio, CI = confidence interval

**Supplementary Table 3B**. Multivariable analysis without TSR demonstrating no independent prognostic value of Stromal Protein Score for disease-free survival.

| **Variable** | **HR** | **95% CI** | **p-value** |
| --- | --- | --- | --- |
| *Stromal Protein Score* | | | |
| Male sex | 0.868 | 0.298 – 2.526 | 0.795 |
| Right-sided colon cancer | 2.734 | 0.955 – 7.831 | 0.061 |
| Tumor Stage | - | - | 0.174 |
| ACT given | 2.433 | 0.561 – 10.545 | 0.235 |
| Stromal Protein Score | 1.344 | 0.602 – 3.005 | 0.471 |

TSR = Tumor-Stroma Ratio, HR = hazard ratio, CI = confidence interval
